# Supplementary material for: Consistent negative response of US crops to high temperatures in observations and crop models
Source: Nat Commun. 2017 Jan 19;8:13931. doi: 10.1038/ncomms13931 (PMC5253679; doi:10.1038/ncomms13931)
Supplement: Supplementary Information — Supplementary Figures, Supplementary Tables, Supplementary Notes and Supplementary References [file ncomms13931-s1.pdf]

Supplementary Information: “Consistent negative response of US crops to high temperatures in observations and crop models”

Supplementary Figures

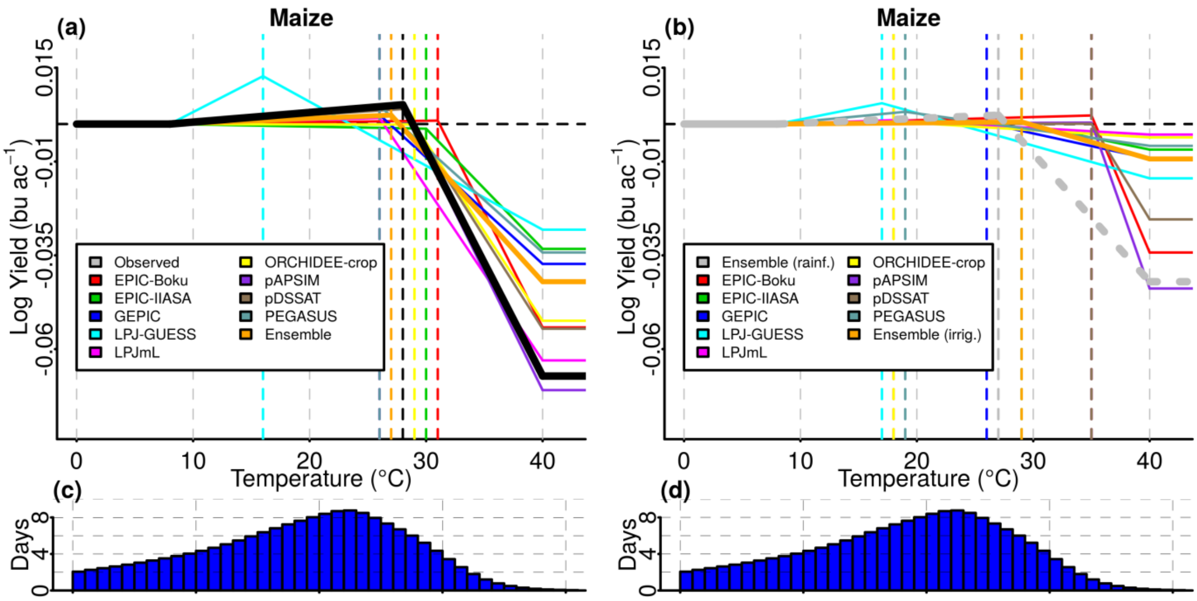

Supplementary Figure 1: Regression of US maize according to the “piecewise linear” approach in rainfed counties. Panels (**a,b**) show regression coefficients and panels **(c,d)** display the temperature exposure during an average, fixed growing season. Yields in panel (**a**) are rainfed while yields in panel (**b**) are irrigated. The rainfed ensemble line is drawn for comparison also in panel **(b)** (grey dashed line). The pattern of yield response to temperature exposure is clearly visible for the rainfed yields: a significantly positive response to intermediate, but a strong negative response to high temperatures, both in observed and simulated yields (panel **a**). For simulated irrigated yields, in contrast, a significant inflection point from high temperature damage is missing (six models + ensemble; panel **b**) or occurs only at higher temperatures and less pronounced (EPIC-Boku, pAPSIM and pDSSAT).

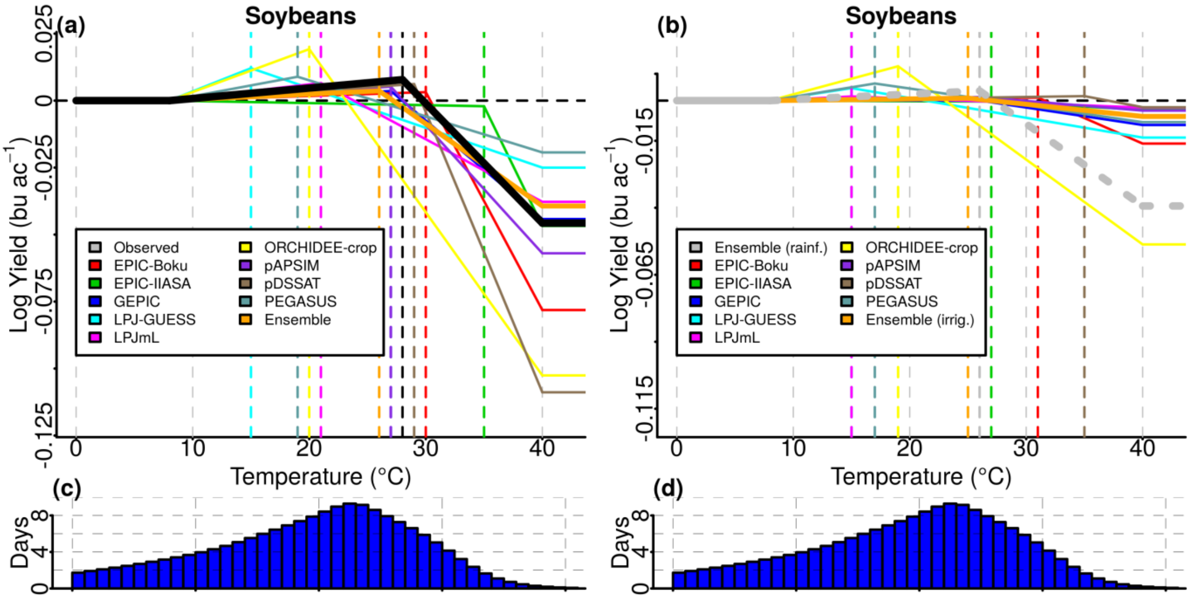

Supplementary Figure 2: Regression coefficients of US soybean according to the "piecewise linear" approach in rainfed counties. Panels and colors are as in Supplementary Figure 1.

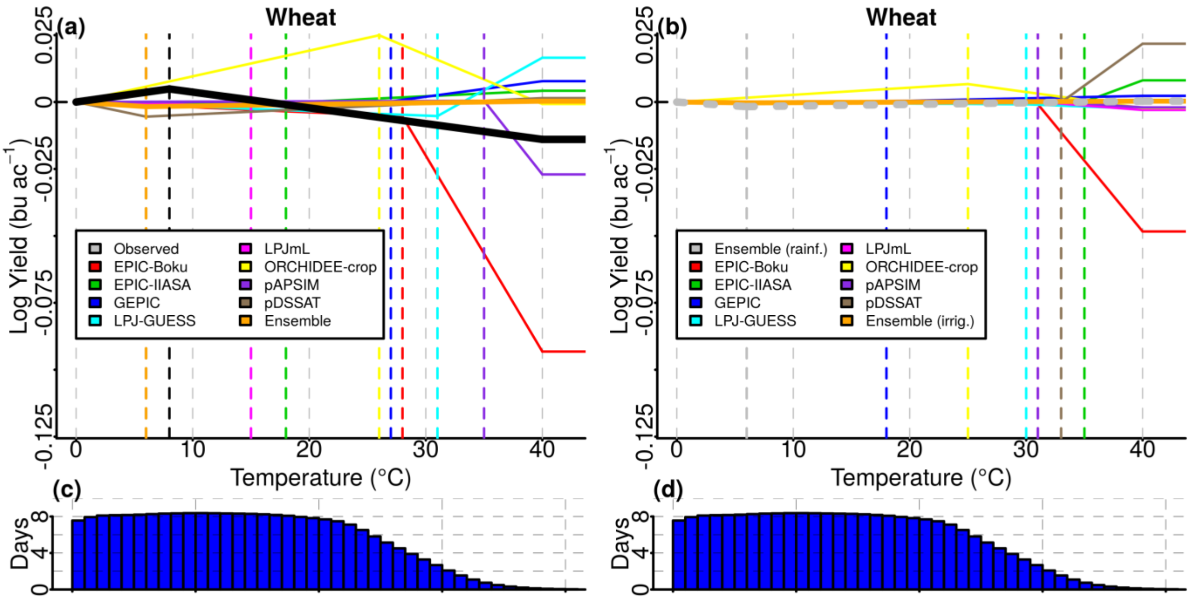

Supplementary Figure 3: Regression of US wheat according to the "piecewise linear" approach in rainfed counties. Panels and colors are as in Supplementary Figure 1.

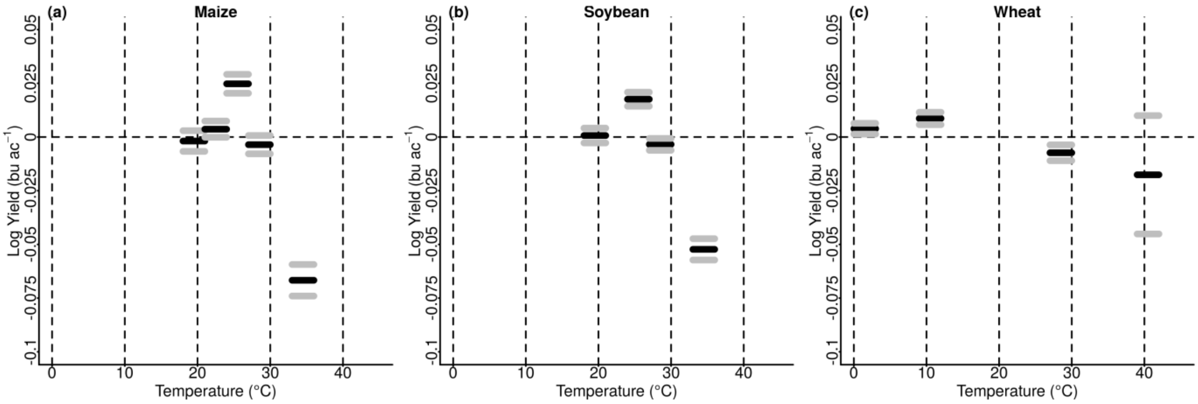

Supplementary Figure 4: Regression analysis for principal temperature components only. Rainfed observed maize (panel **a**), soybean (panel **b**) and wheat (panel **c**) show the same responses as with the full regression frame. Black lines show coefficients and grey lines show 95%-confidence intervals.

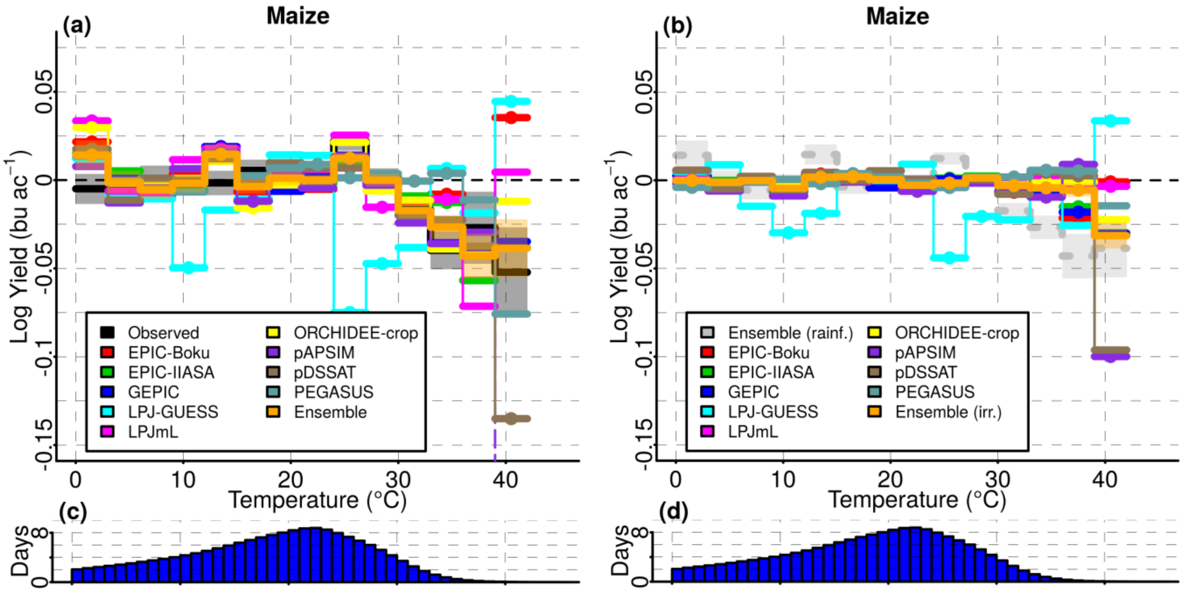

Supplementary Figure 5: Regression coefficients for **(a)** rainfed and **(b)** irrigated simulated maize. The black curve in panel **(a)** shows the observed yield response, while the grey curve in panel **(b)** shows the *simulated rainfed* ensemble response for comparison. The simulation runs were performed under the ‘harmnoN’ scenario (see text) in rainfed counties. Panels **(c,d)** show temperature exposures during an average, fixed growing season. Colored lines indicate different models. More details about the two simulation scenarios can be found in ref.[1]. Results are shown for the ‘fixed’ growing season, but are not qualitatively different for the model-specific growing seasons (data not shown).

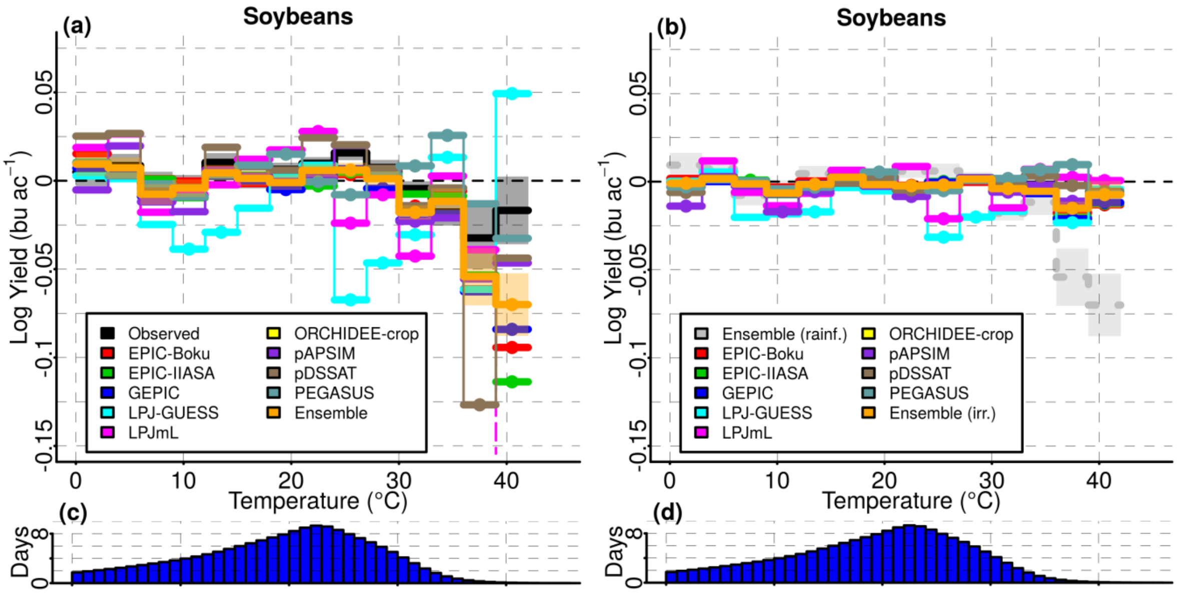

Supplementary Figure 6: Regression coefficients for **(a)** rainfed and **(b)** irrigated simulated soybean under the 'harmnoN' scenario. Panels **(c,d)** show temperature exposures during an average, fixed growing season. Colors are as in Supplementary Figure 5.

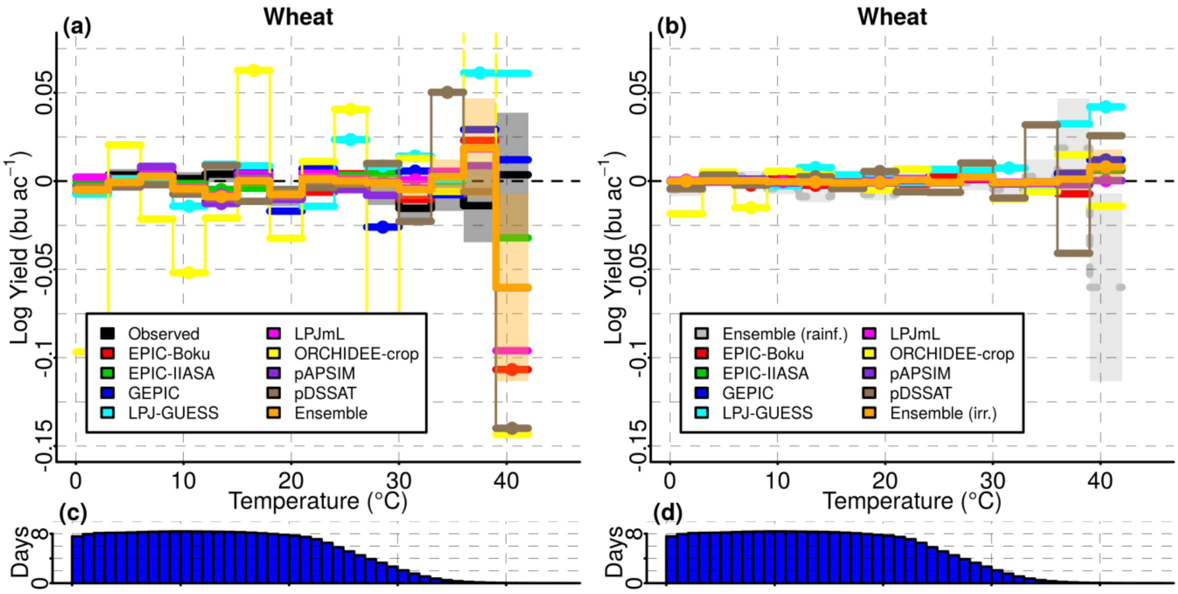

Supplementary Figure 7: Regression coefficients for **(a)** rainfed and **(b)** irrigated simulated wheat under the 'harmnoN' scenario. Panels **(c,d)** show temperature exposures during an average, fixed growing season. Colors are as in Supplementary Figure 5.

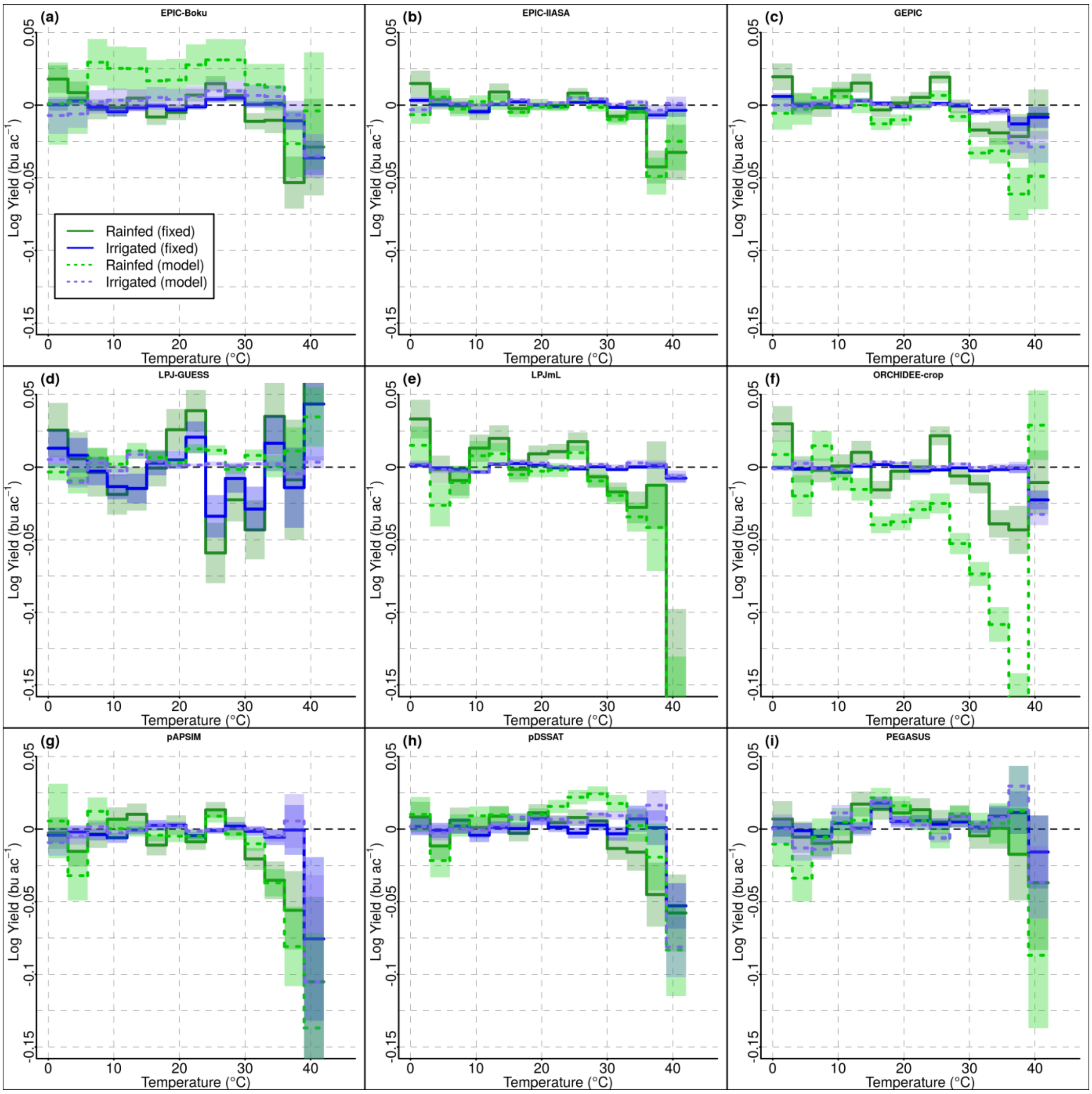

Supplementary Figure 8: Regression coefficients for US maize from the nine individual crop models used in our ensemble. For each model four setups are analyzed: rainfed with fixed (March 01 – August 31) growing season (solid green) or model-calculated growing season (dashed green), and irrigated with fixed (solid blue) or model dates (dashed blue). Shaded areas are 95% confidence intervals. A note on LPJ-GUESS: the low average yield amount simulated by LPJ-GUESS (in the considered region) inherently increases yield variability; this may lead to a reduced signal-to-noise ratio, which is the likely reason behind the unique temperature response of this model.

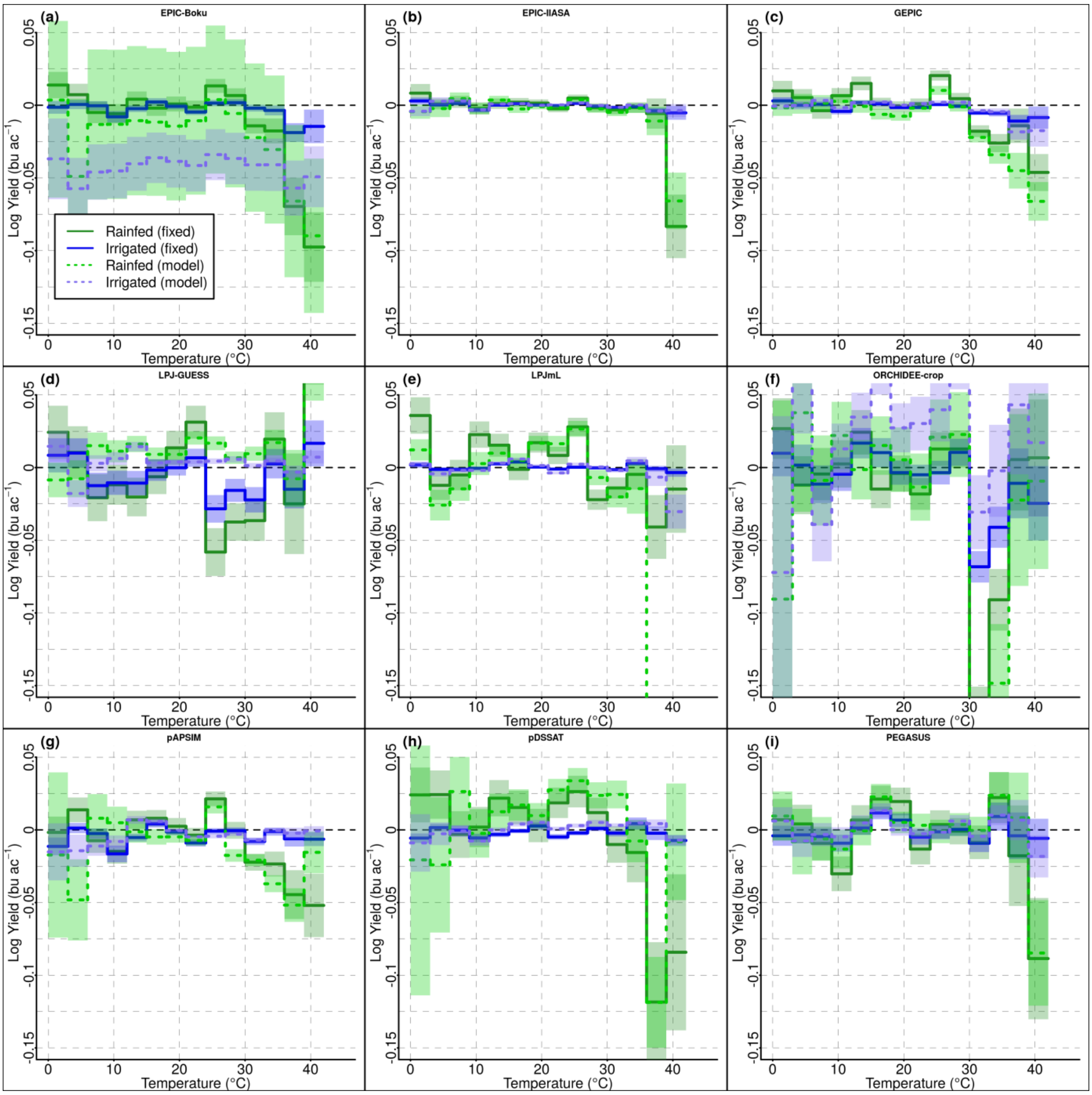

Supplementary Figure 9: Regression coefficients for US soybean from the nine individual crop models used in our ensemble. Colors are as in Supplementary Figure 8. For LPJ-GUESS and ORCHIDEE-crop the same arguments apply as for maize.

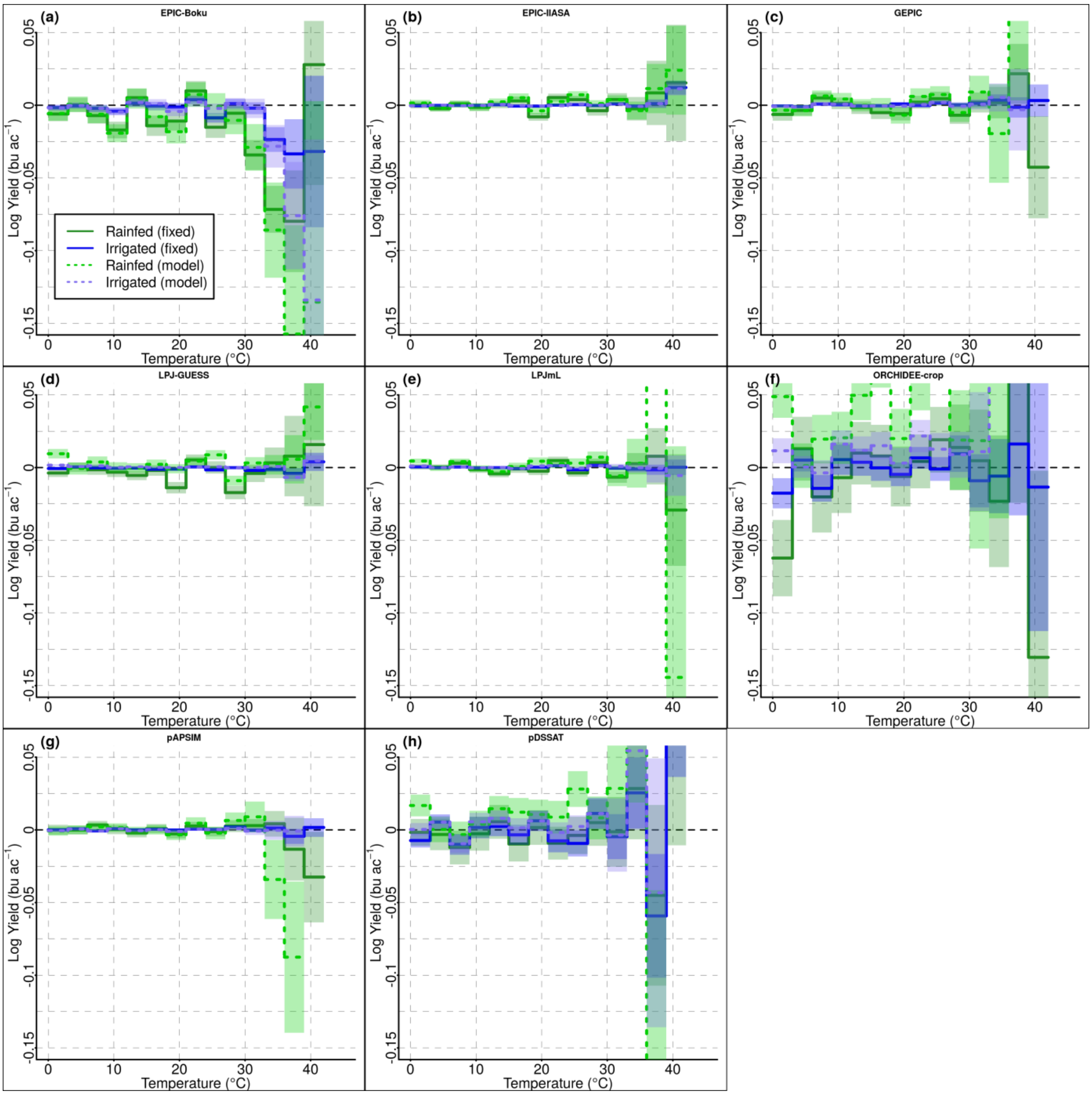

Supplementary Figure 10: Regression coefficients for US wheat from the nine individual crop models used in our ensemble. Colors are as in Supplementary Figure 8.

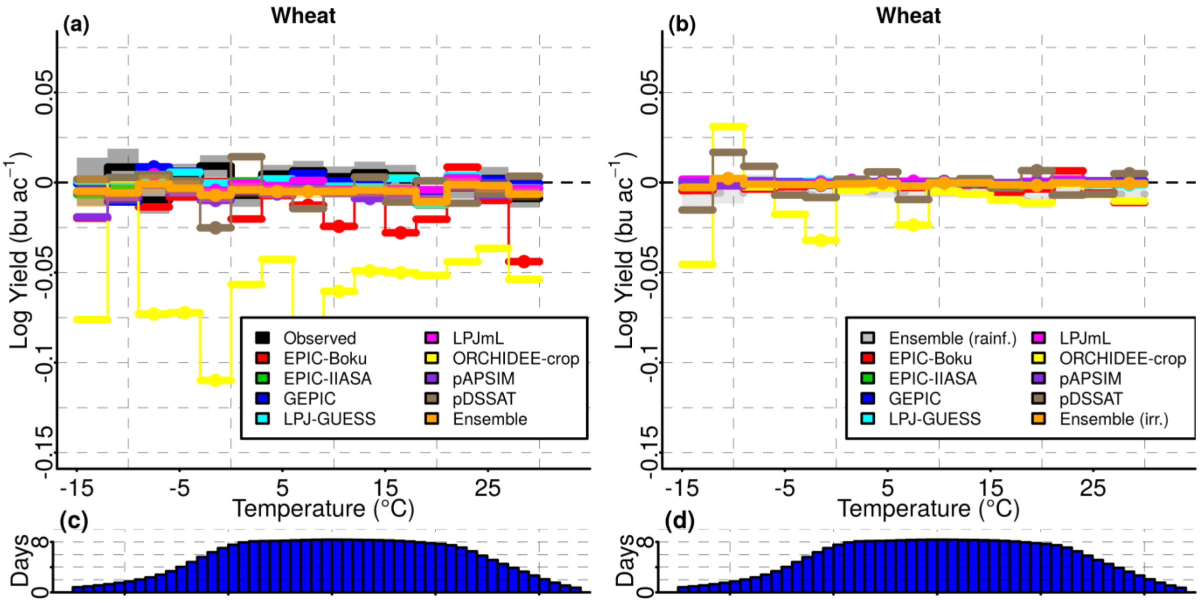

Supplementary Figure 11: Wheat response to temperature, with a broader temperature range down to -15°C, in rainfed counties. Panels **(a,b)** show yield responses to different temperature bins with **(a)** rainfed or **(b)** irrigated simulations. Panels **(c,d)** show temperature exposures during an average, fixed growing season. Colored lines represent individual models. The grey dashed line in panel **(b)** is the simulated rainfed ensemble response for comparison (orange line in panel **a**).

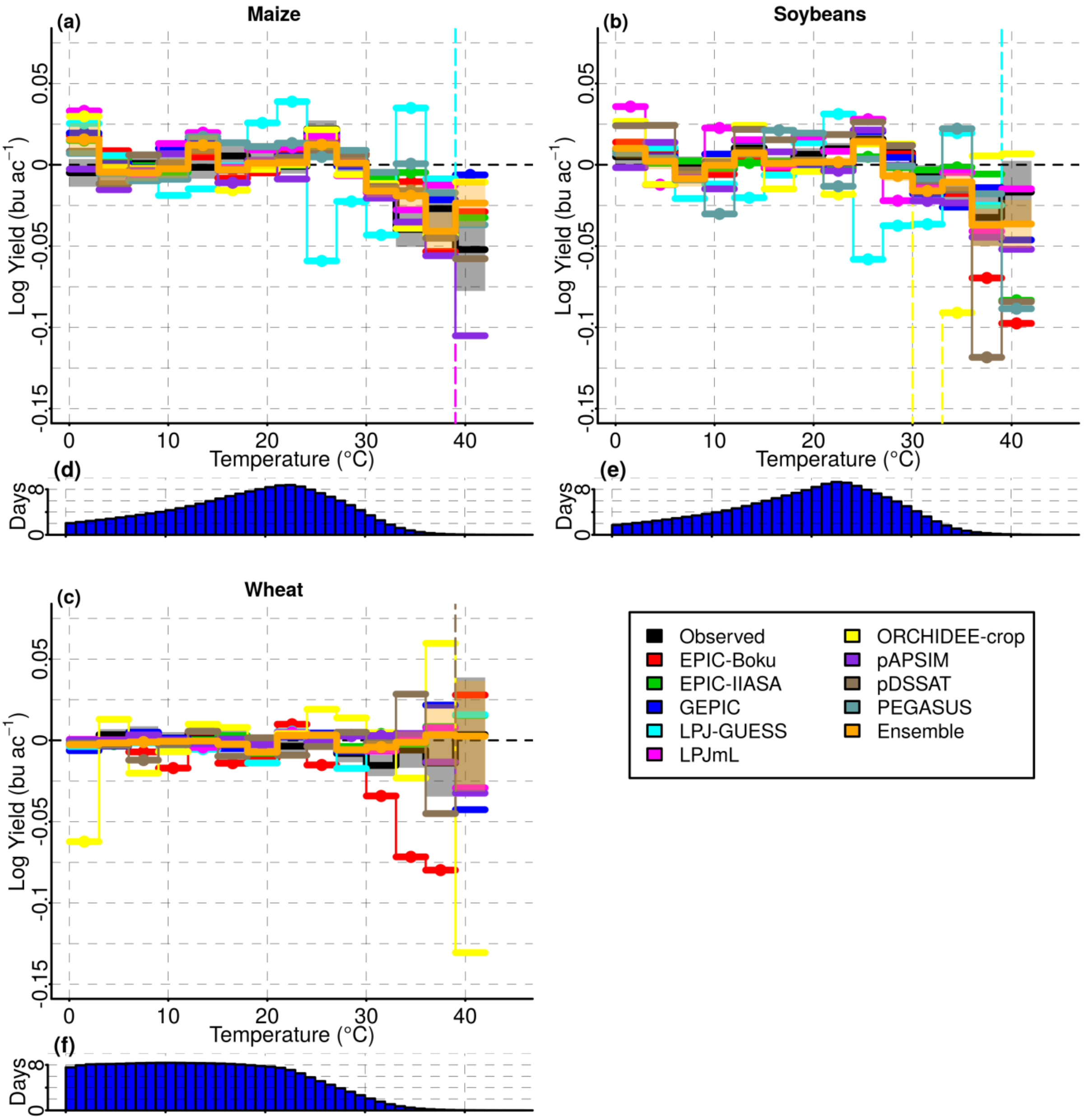

Supplementary Figure 12: Comparison of simulated to observed effects of high temperatures on rainfed yields in rainfed counties. Panels **(a-c)** show coefficients for **(a)** maize, **(b)** soybean and **(c)** wheat. Panels **(d-f)** show the mean temperature exposure over the analyzed area, averaged over all years. Black lines in panels **(a-c)** are coefficients (γ_h) for log observed yield if the crop is exposed for one day to a particular 3°C temperature interval. Colored lines are coefficients for the simulated yields (orange = ensemble median). Estimates are derived by a panel regression (equation 1) of US county data where the considered crop is grown under predominantly (> 90%) rainfed conditions. Grey and orange shaded areas represent 95% confidence intervals. Coefficients for observed yields significantly differing from 0 are marked with a black dot. Simulated coefficients are marked by colored dots if they are significantly different from the observed coefficients (confidence intervals do not overlap). The analysis is based on the assumption of a fixed growing season following ref. [2].

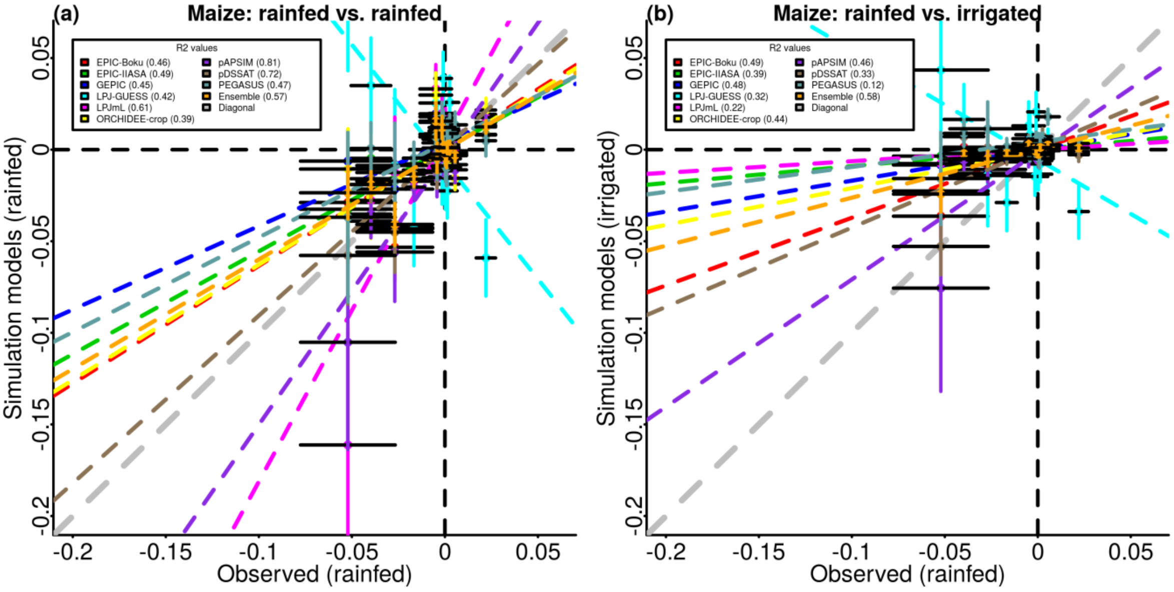

Supplementary Figure 13: Correlation plots of temperature coefficients for simulated rainfed (panel **a**) and irrigated (panel **b**) vs. observed rainfed maize in the US, all for rainfed counties. On the x-axis the coefficients for the regression with rainfed observed yields are shown, while on the two y-axes the coefficients of the different crop models are displayed. In panel **(a)** both observed and simulated yields are rainfed, while in panel **(b)** the observed yields are still rainfed, but the simulated ones are irrigated. Different colors denote different models, and numbers in brackets in the legend indicate the R^2 for each model-to-observed linear correlation of coefficients. The lines around points are 95% confidence intervals. Gray dashed lines are 1:1 lines for comparison.

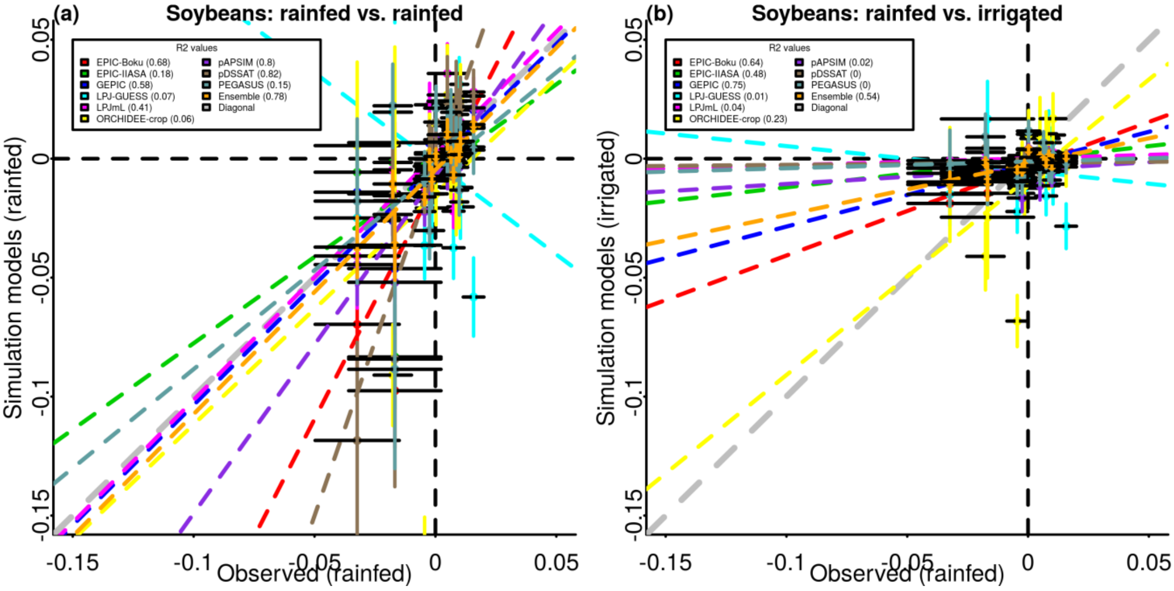

Supplementary Figure S14: Correlation plots of temperature coefficients for simulated rainfed (panel **a**) and irrigated (panel **b**) vs. observed rainfed soybean in US rainfed counties. Colors are as in Supplementary Figure 13.

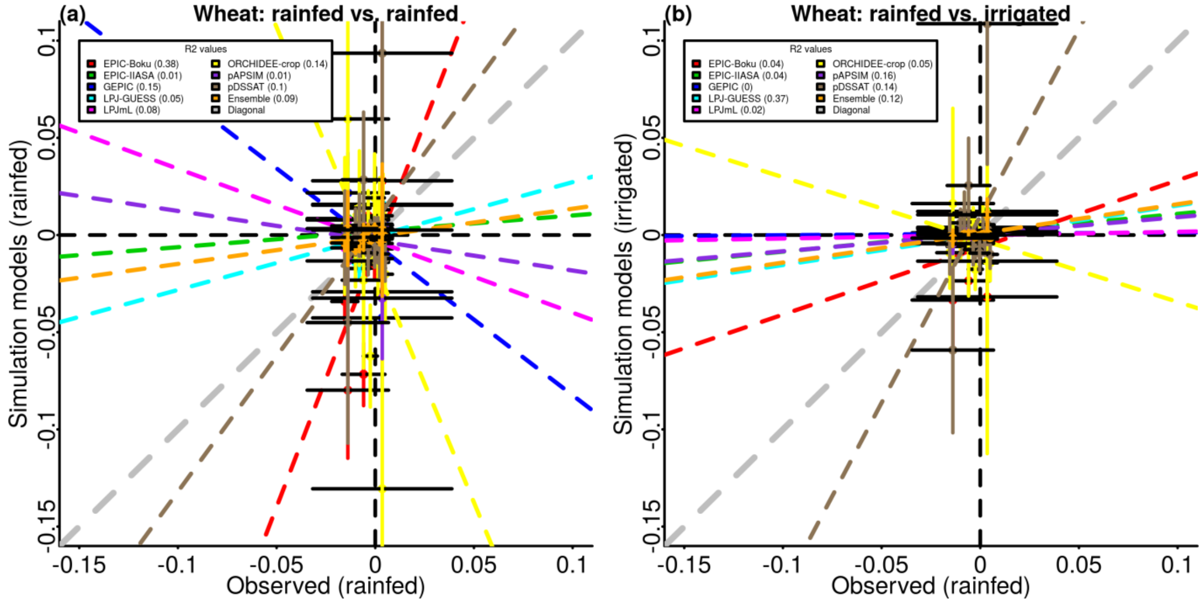

Supplementary Figure 15: Correlation plots of temperature coefficients for simulated rainfed (panel **a**) and irrigated (panel **b**) vs. observed rainfed wheat in US rainfed counties. Colors are as in Supplementary Figure 13. There is no pattern in either of the two water supply scenarios, indicating that temperature-induced water stress does not play a major role for historical wheat yields. Negative slopes can occur spuriously from a clustering of the coefficients around 0 with large confidence intervals.

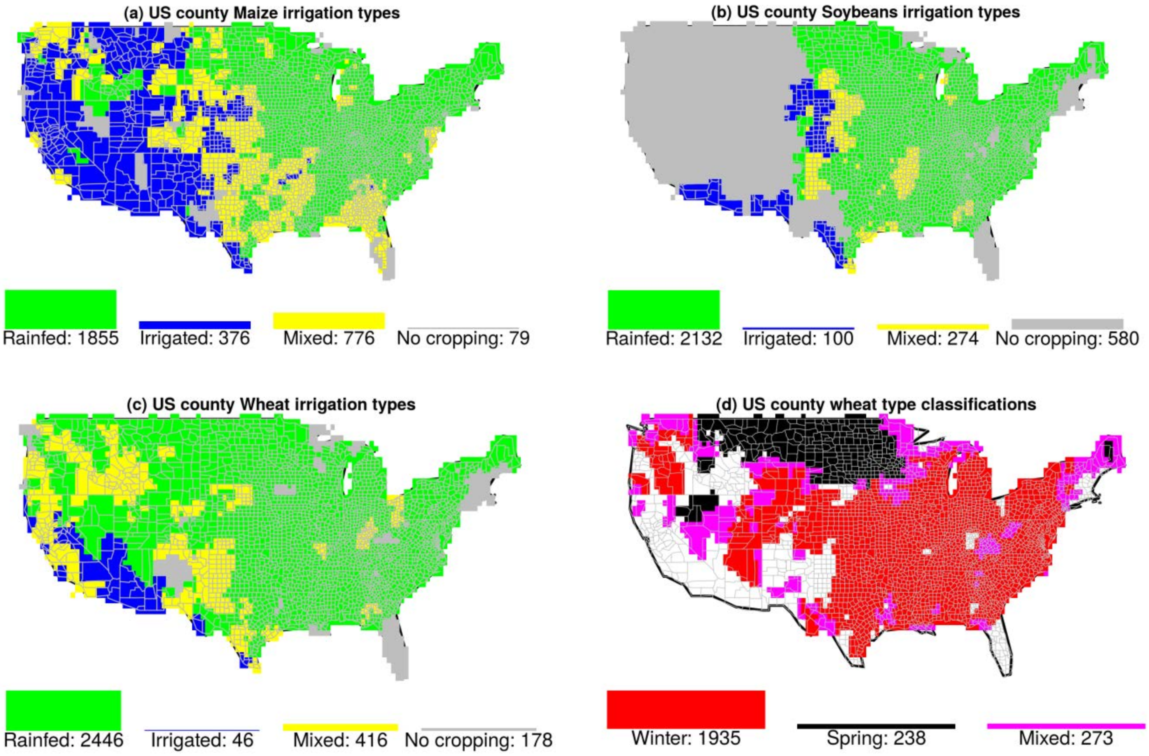

Supplementary Figure 16: US county irrigation classifications for maize **(a)**, soybean **(b)** and wheat **(c)**. The type of rainfed wheat is indicated in panel **(d)**; a threshold of 90% is used to define purely winter or spring wheat counties, respectively. Numbers below the histograms are county counts (of 3,086 in total). Counties were classified as ‘rainfed’ or ‘irrigated’ if the crop-specific share of agricultural practice in this county was at least 90% (rainfed) or 75% (irrigated), respectively; all others were classified as ‘mixed’. Counties with no harvested area of the respective crop are stated as ‘No cropping’.

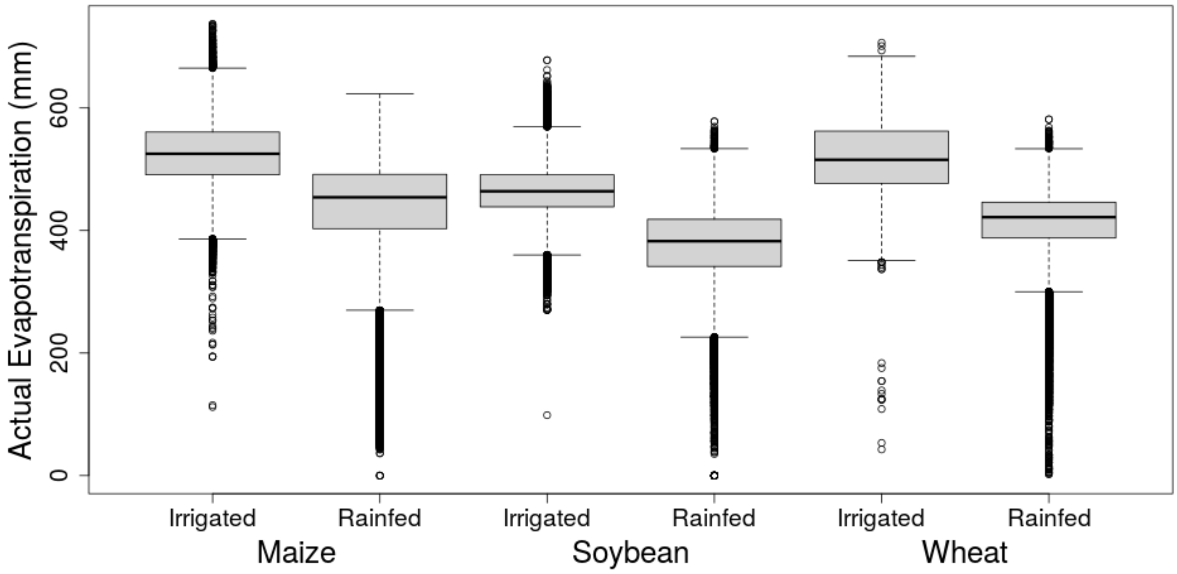

Supplementary Figure 17: Actual evapotranspiration over the historical growing season for the three crops maize, soybean and wheat under irrigated and rainfed conditions. All pairwise t-tests for mean difference are highly significant (p = 0); relative differences are shown in Supplementary Table 3.

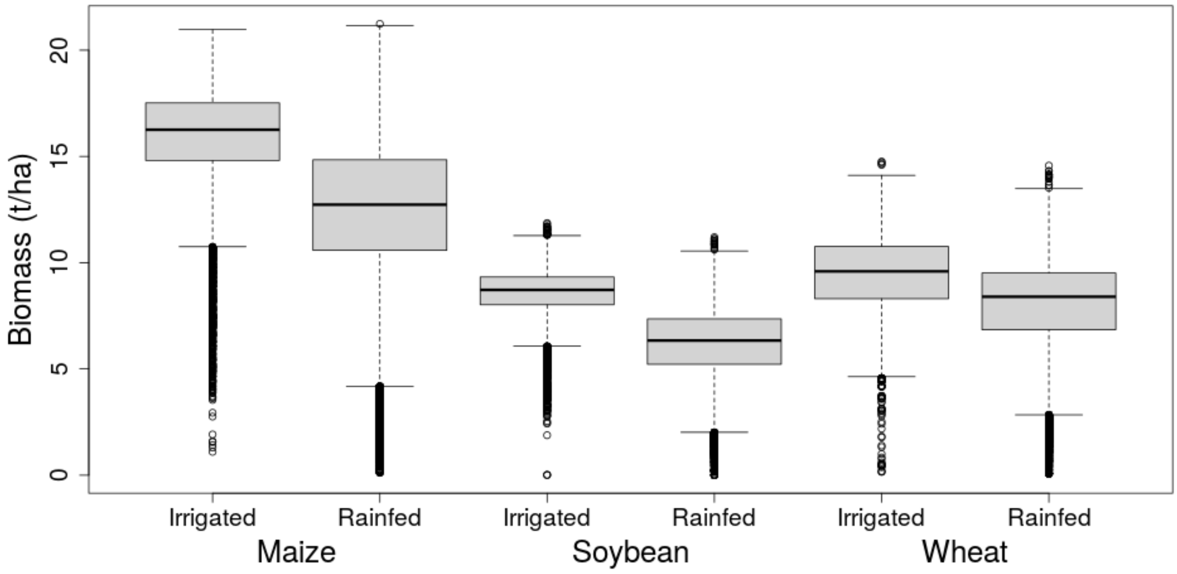

Supplementary Figure 18: Biomass accumulation over the historical growing season for the three crops maize, soybean and wheat under irrigated and rainfed conditions. All pairwise t-tests for mean difference are highly significant (p = 0); relative differences are shown in Supplementary Table 3.

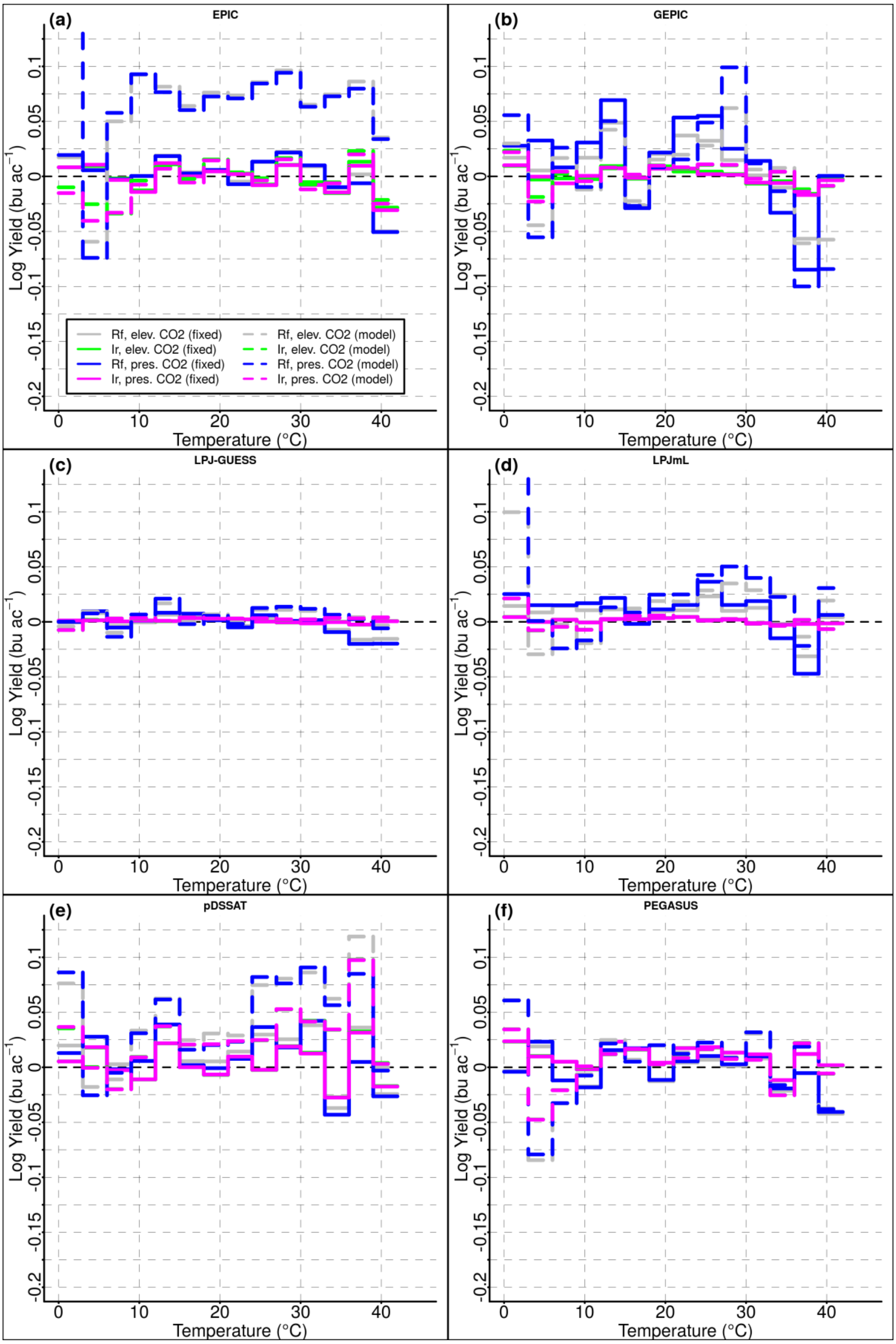

Supplementary Figure 19: Regression results for the future simulations from individual models of US maize in rainfed counties. Panels are EPIC-Boku **(a)**, GEPIC **(b)**, LPJ-GUESS **(c)**, LPJmL **(d)**, pDSSAT **(e)** and PEGASUS **(f)** models, respectively. Growing season has either been fixed from March 01 to August 31 ('fixed') or been taken from the simulation models ('model'). Confidence intervals are not drawn for visual clarity.

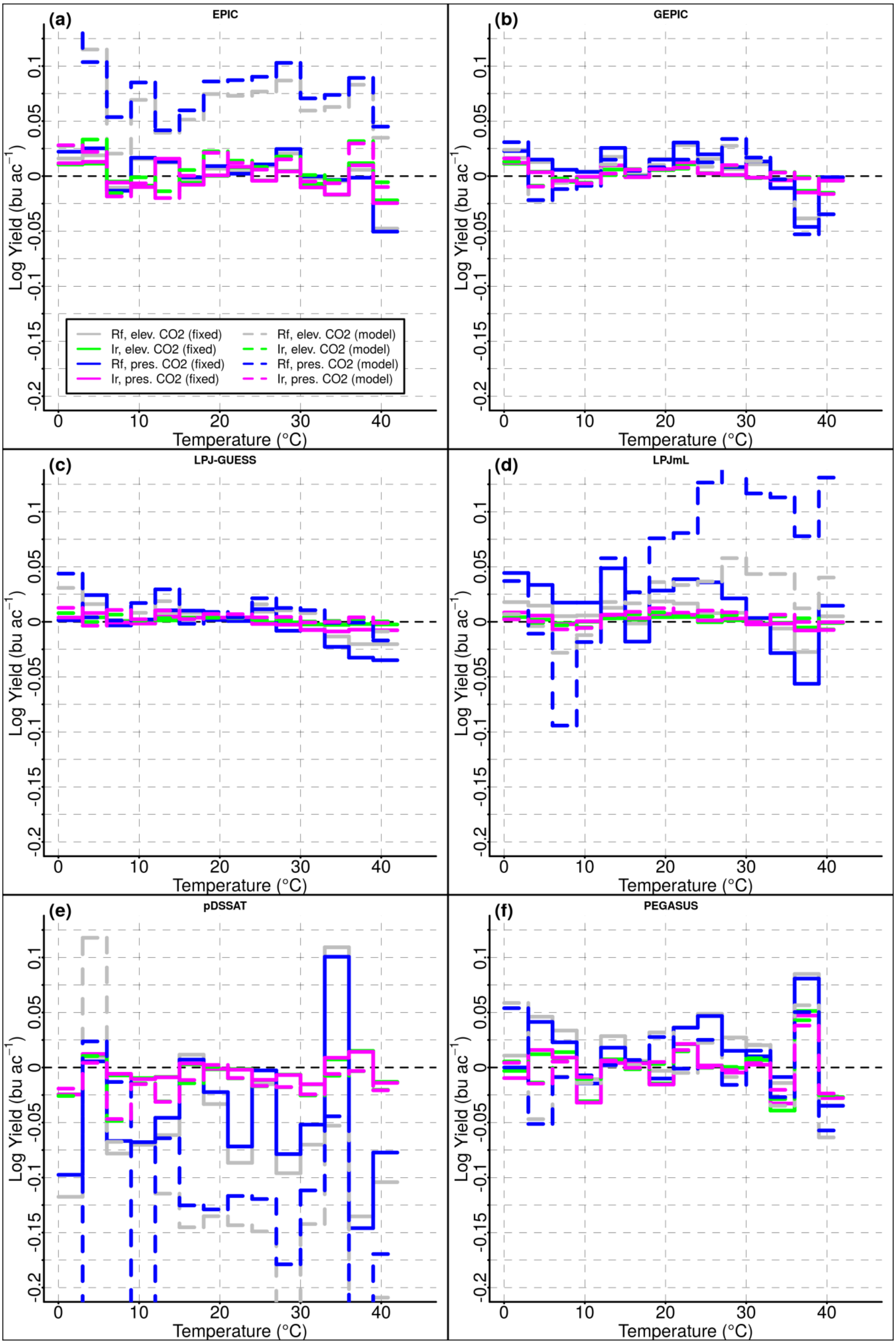

Supplementary Figure 20: Regression results for future simulations from individual models of US soybean in rainfed counties. Colors are as in Supplementary Figure 19.

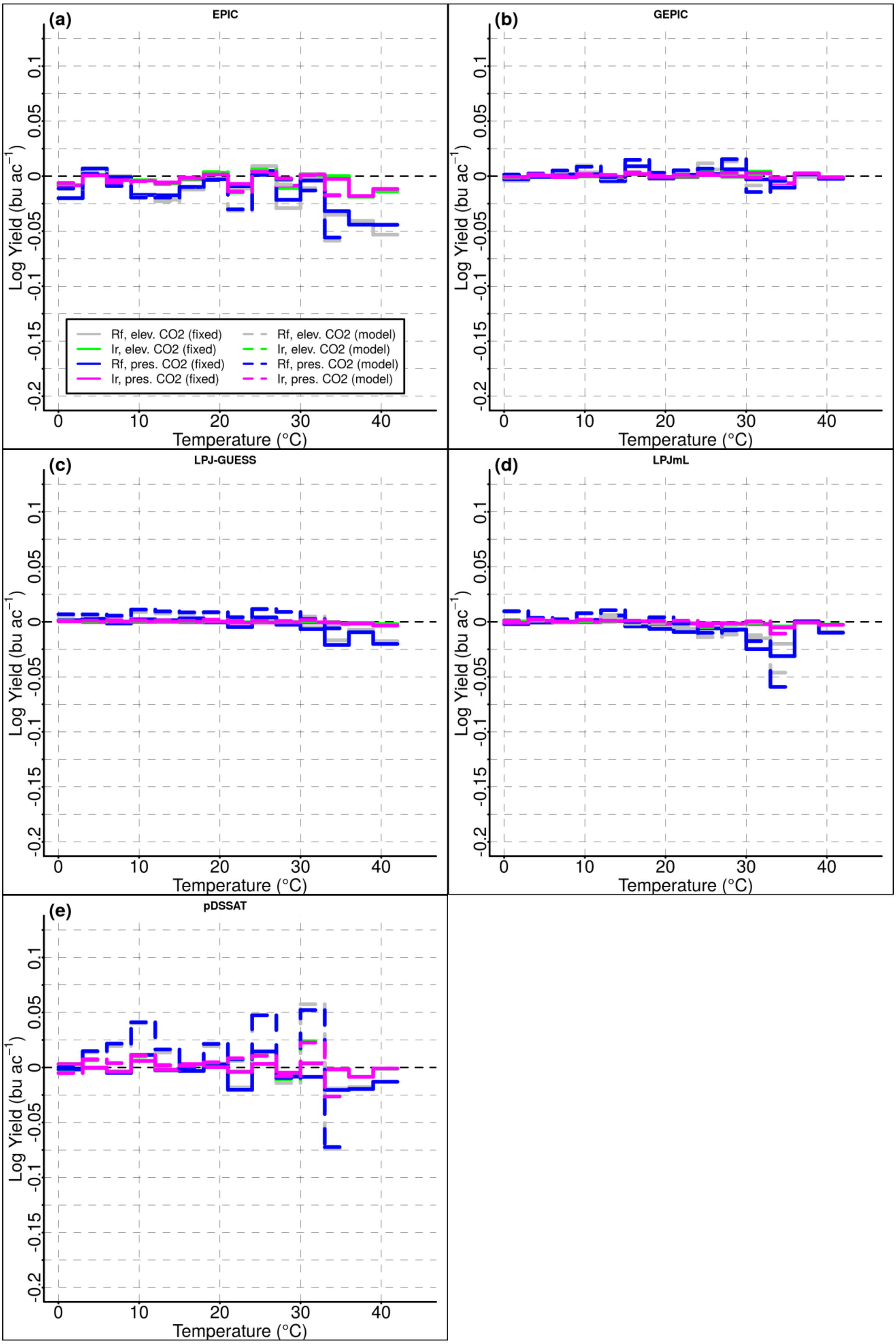

Supplementary Figure 21: Regression results for future simulations from individual models of US wheat in rainfed counties. Colors are as in Supplementary Figure 19.

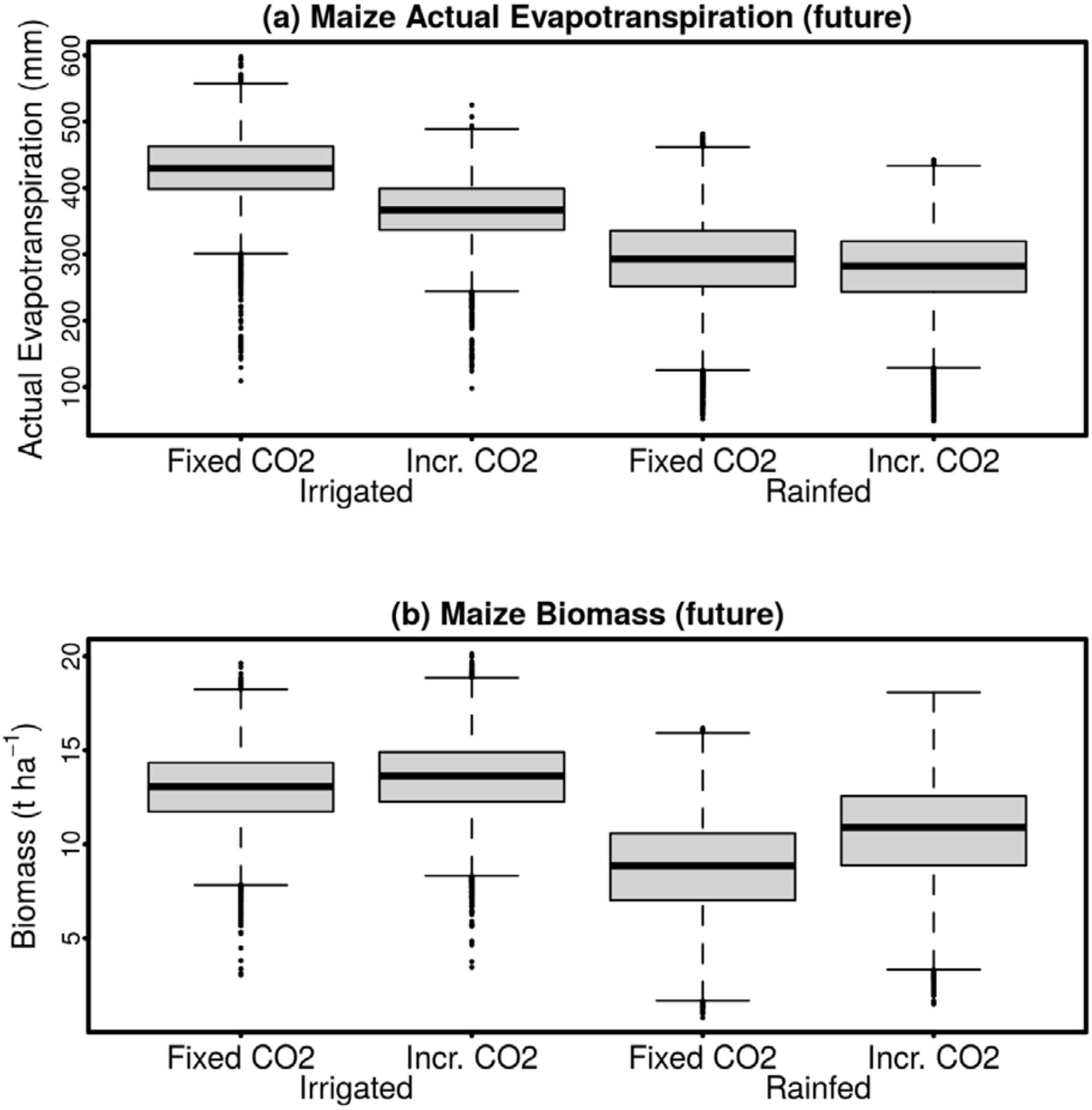

Supplementary Figure 22: Actual evapotranspiration **(a)** and biomass **(b)** over the future growing seasons for maize under four different irrigation (irrigated/rainfed) and [CO_2] (fixed present/increased) combinations. All pairwise t-tests for mean difference are highly significant ($p = 0$); relative differences are shown in Supplementary Table 4.

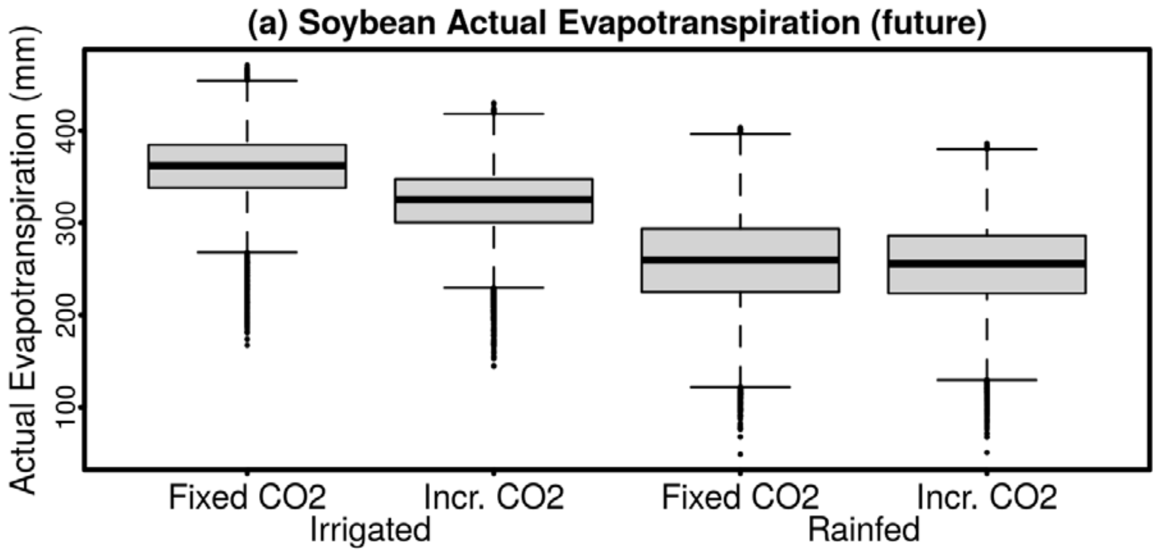

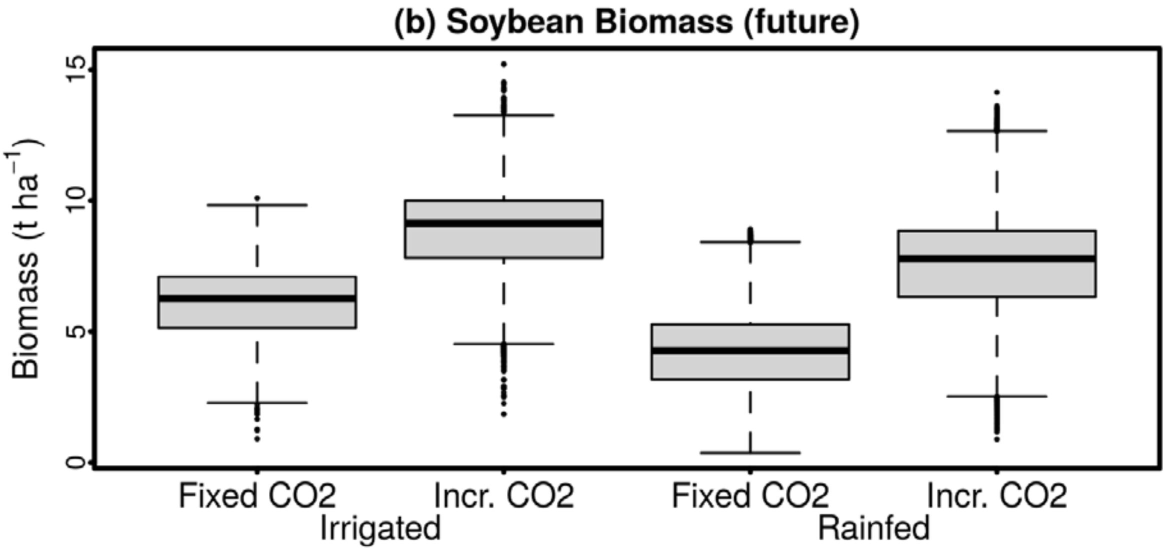

Supplementary Figure 23: Actual evapotranspiration **(a)** and biomass **(b)** over the future growing seasons for soybean under four different irrigation (irrigated/rainfed) and [CO_2] (fixed present/increased) combinations. All pairwise t-tests for mean difference are highly significant ($p = 0$); relative differences are shown in Supplementary Table 4.

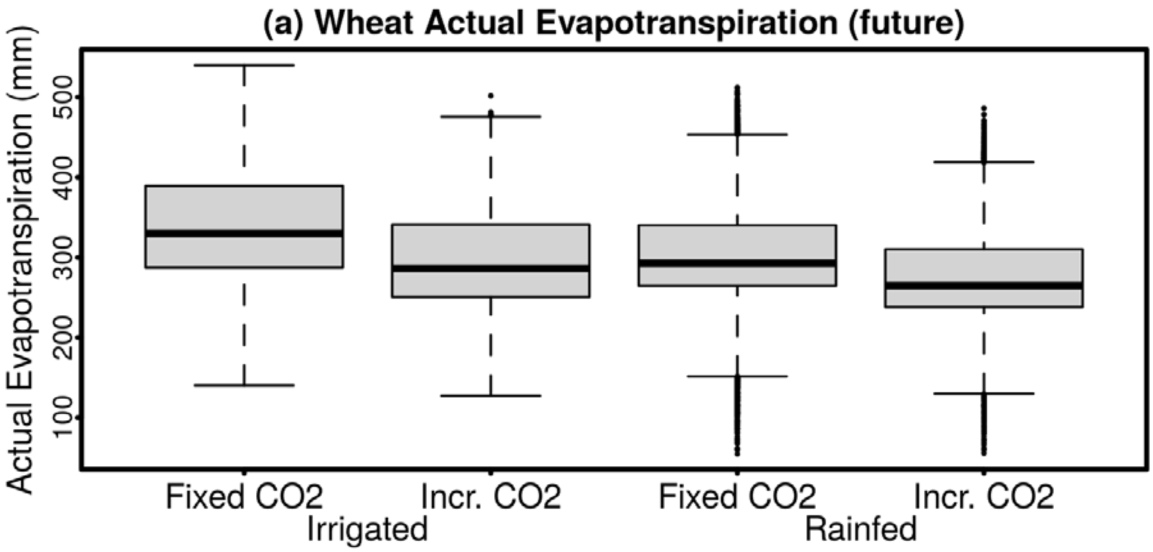

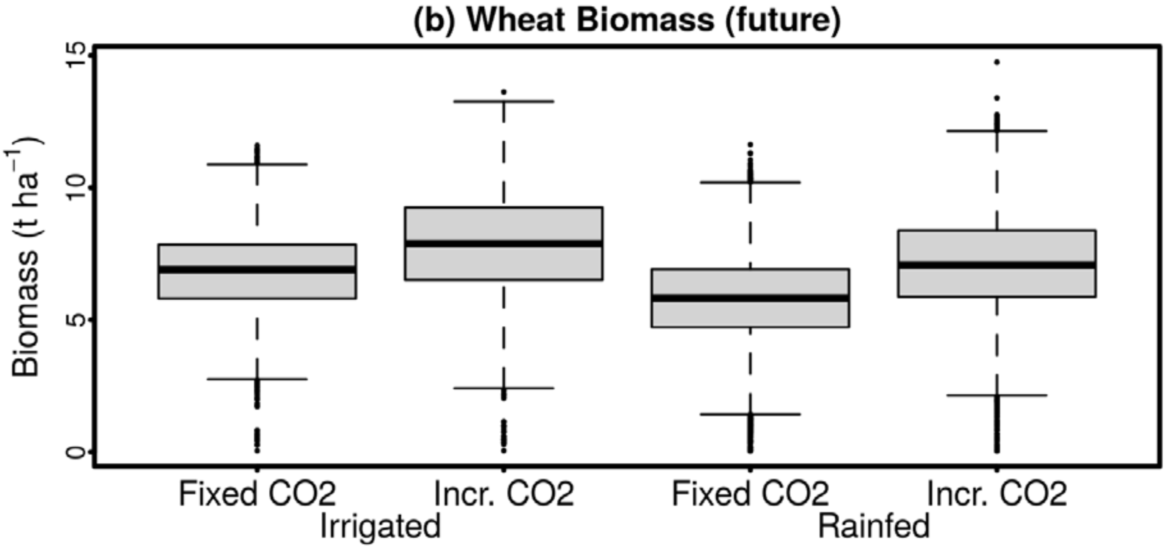

Supplementary Figure 24: Actual evapotranspiration **(a)** and biomass **(b)** over the future growing seasons for wheat under four different irrigation (irrigated/rainfed) and [CO_2] (fixed present/increased) combinations. All pairwise t-tests for mean difference are highly significant (p = 0); relative differences are shown in Supplementary Table 4.

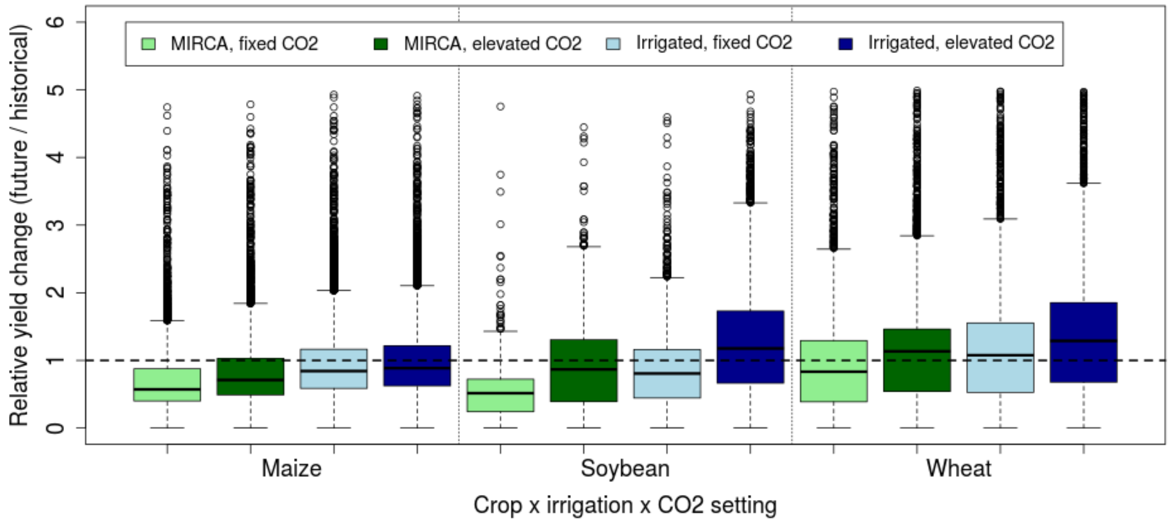

Supplementary Figure 25: Relative changes in time-averaged county yields between future and historical periods. Comparisons are individual for each crop model, but summarized in boxplots. A value of 1.0 (horizontal dashed line) indicates no change. “MIRCA” is the current irrigation pattern, and “Irrigated” is full irrigation on all cultivated areas. Outliers above 5 were removed for visual clarity (0.4% of the data). Only counties were considered where yields were available for both historical and future simulations (removed 24% of the data).

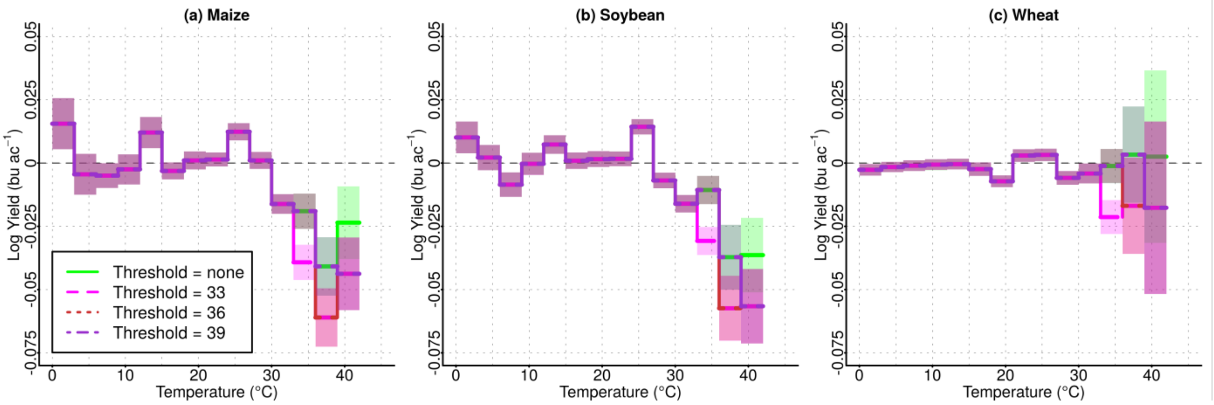

Supplementary Figure 26: Sensitivity of the statistical model to artificial yield losses from extremely high temperatures. Panels are maize **(a)**, soybean **(b)** and wheat **(c)**. Shaded areas are 95% confidence intervals. Different colors denote different temperature thresholds for yield reduction. Green curves (no reduction) are equal to green curves in Figure 1 of the main paper.

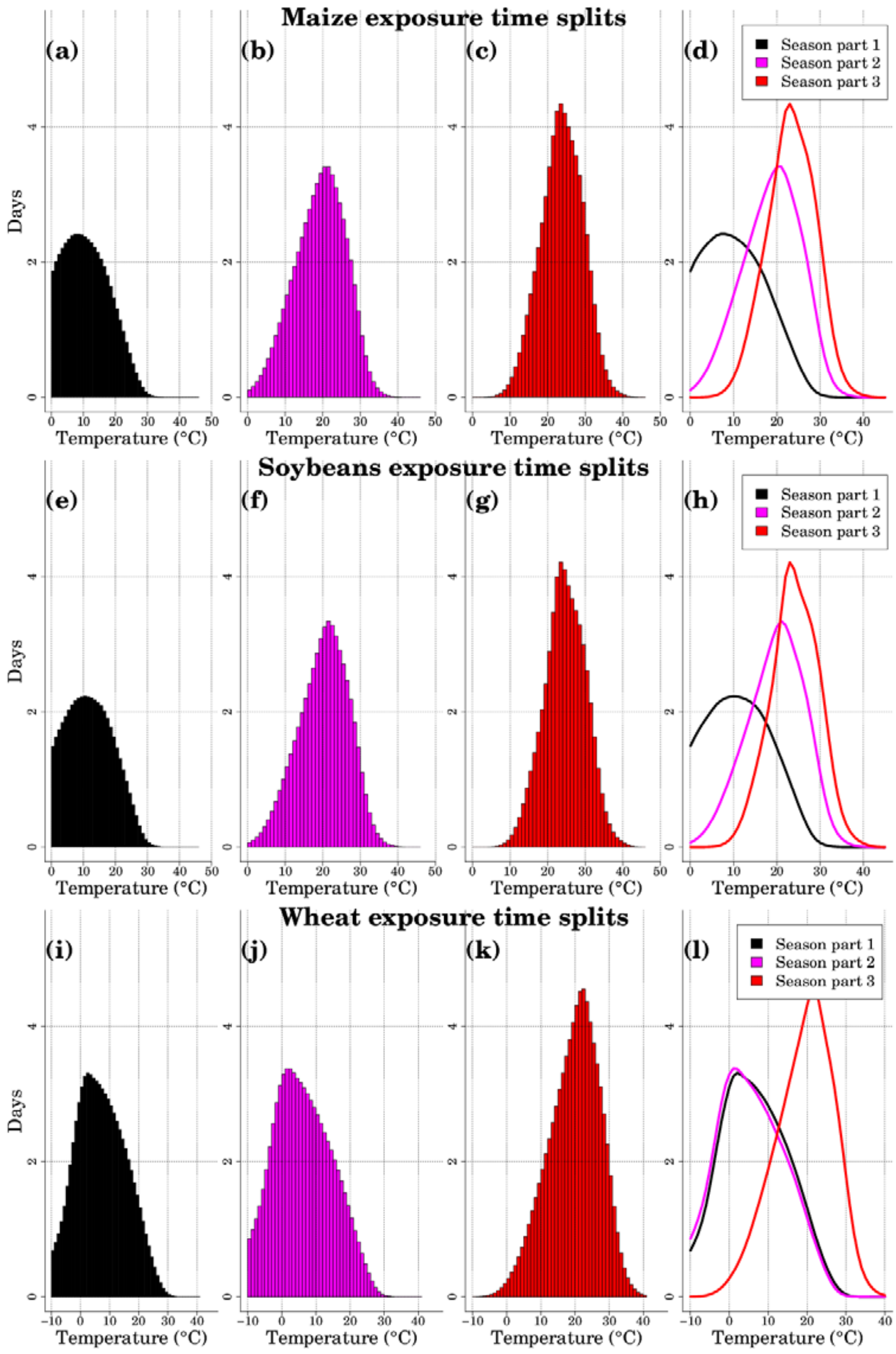

Supplementary Figure 27: Exposure times to 1°C bins during different parts of the historical fixed growing season. Panels show maize **(a-d)**, soybean **(e-h)** and wheat **(i-l)** exposure time distributions. Panels **a-c, e-g, i-k** display the temperature exposure in days for each third of the growing season . The three histograms are combined in panels **d,h,l**. The crop-specific fixed growing season is split into three equally sized parts. For maize and soybean these are March-April (part 1), May-June (part 2) and July-August (part 3). For wheat the parts are October-January (part 1), January-April (part 2) and April to July (part 3); months are split on day 15 as the fixed winter growing season is from October 15 to July 15.

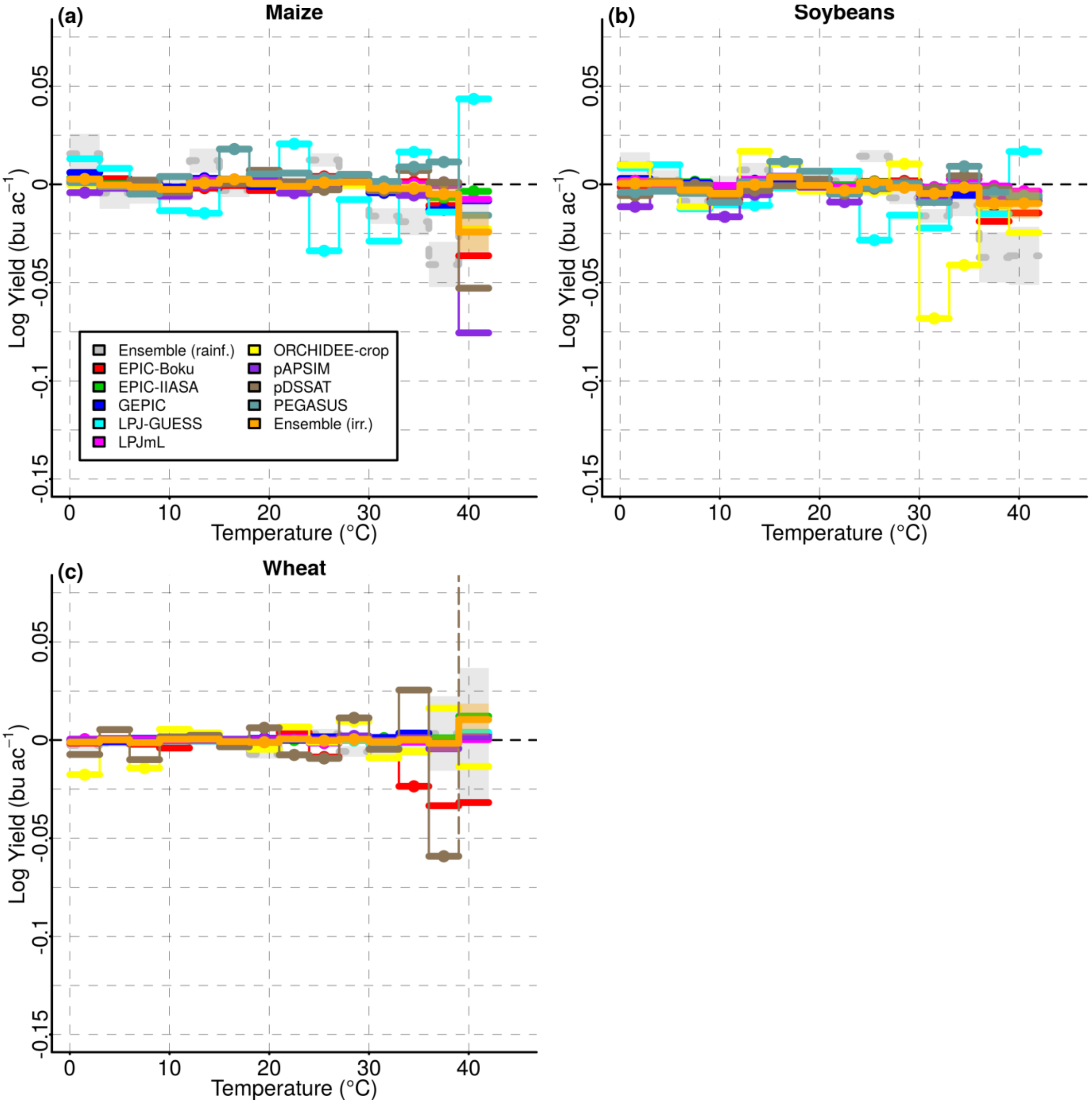

Supplementary Figure 28: Regression coefficients for US yields of individual models. Panels are **(a)** maize, **(b)** soybean and **(c)** wheat. Only US counties with predominantly rainfed agriculture are considered, but simulated yields are <u>fully irrigated</u> (colored lines). The dashed grey line shows coefficients from the 'rainfed' simulation ensemble (not from the observed yields) for comparison. Colored lines denote different models; the orange line is the irrigated ensemble.

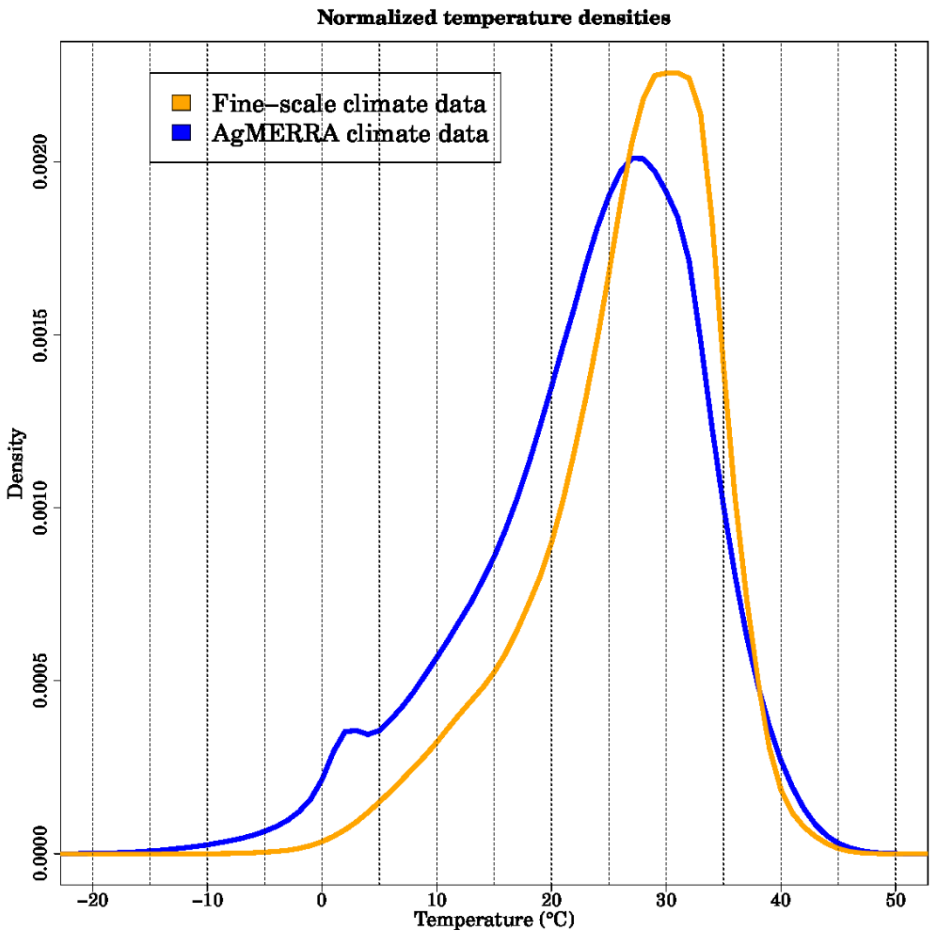

Supplementary Figure 29: Normalized frequency distribution of daily maximum temperatures as derived from the two observational climate data sets used in this study (yellow: temperature data used in the original study by Schlenker & Roberts[2] with a spatial resolution of about 0.04° x 0.04°; blue: temperature data from the AgMERRA data set used in our study and applied to force the crop model simulations with a spatial resolution of 0.5° x 0.5°). The distributions are based on the sample of all daily maximum temperatures across all grid cells without spatial or temporal aggregation. No land-use weighting has been applied.

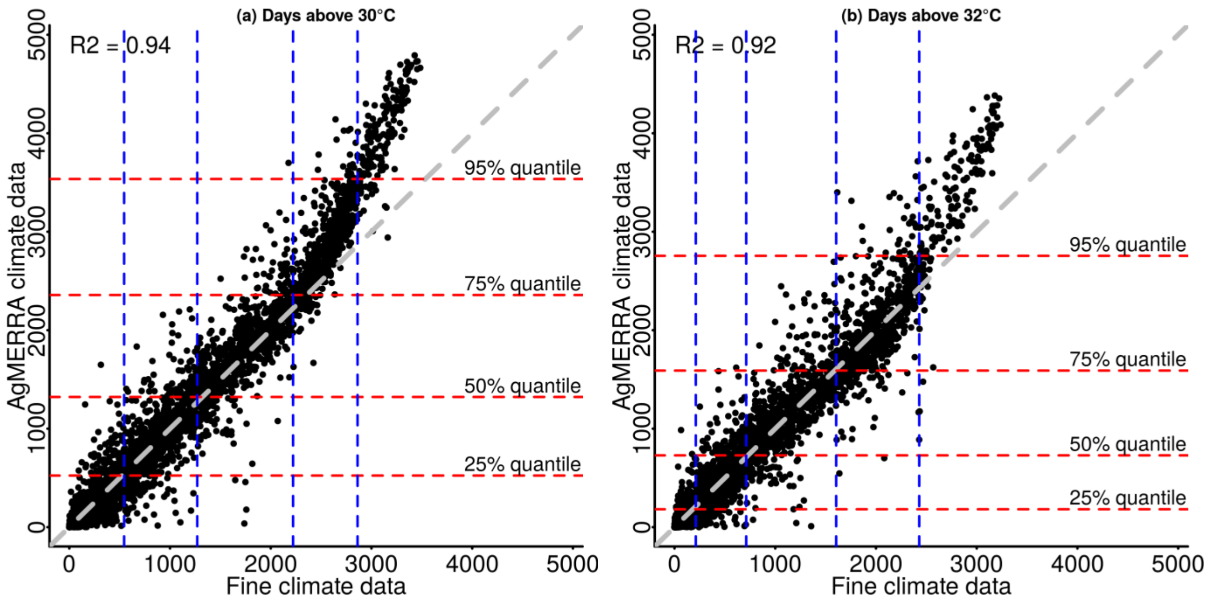

Supplementary Figure 30: Comparison of days with maximum temperature above 30°C (panel **a**) or 32°C (**b**) in all growing seasons from 1980 to 2010 for both data sets in the whole US. The x axis contains the number of days for the fine-scale climate data, while the y-axis contains the corresponding number of days for the AgMERRA climate data. Each dot corresponds to one 0.5° spatial grid cell. Red dashed lines indicate quantiles derived from the AgMERRA climate data and blue lines for the fine-scale climate data. The R^2 values in the top left corner indicate the squared correlation coefficient. Day counts for the fine-scale climate data have been computed for each 2.5-mile grid cell and then this number has been averaged within each 0.5° grid cell.

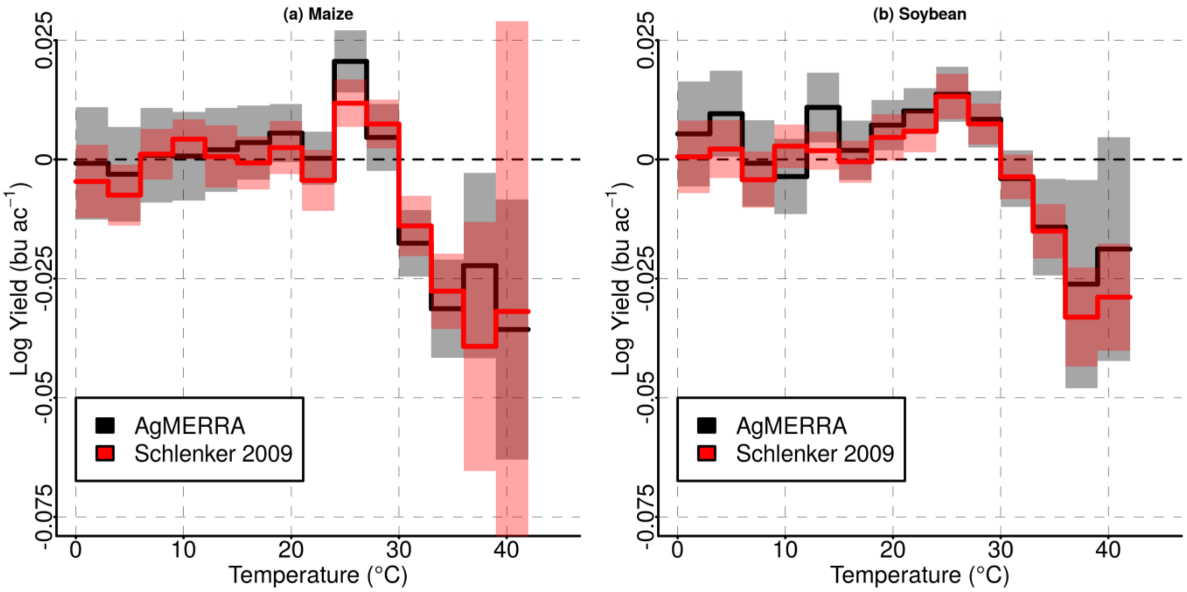

Supplementary Figure 31: Comparison of yield responses to temperature at different spatial resolutions. Maize is shown in panel **(a)** and soybean in panel **(b)**. Red lines: Temperature-bin specific coefficients γ as derived by Schlenker & Roberts[2] from the panel of all US counties east of the 100° meridian based on very high resolution temperature data (similar to Figure 1 of their paper). Black lines: Analogous analysis of the same panel data but based on the lower resolution AgMERRA data. Shaded areas are 99.5% confidence intervals.

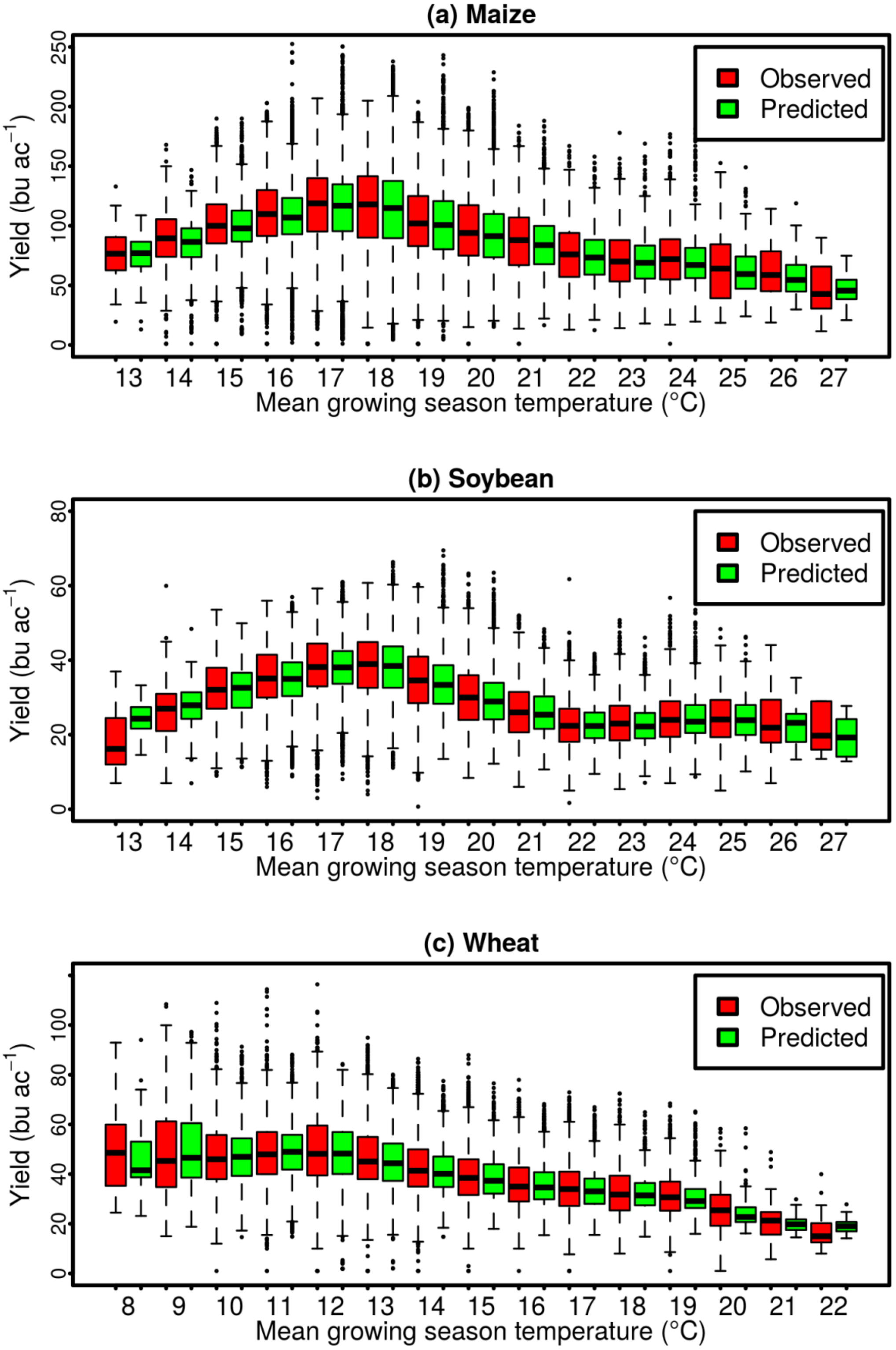

Supplementary Figure 32: Comparison of observed and predicted yields from the regression model against mean growing season temperature. Panels are rainfed maize (a), soybean (b) and wheat (c). Observed yields are shown in red, while predicted yields are shown in green. The box plots show the median (black line within the box) and the first and third quartile (boxes). Whiskers extend to approx. the 1.6-times interquartile range and outliers are drawn with circles.

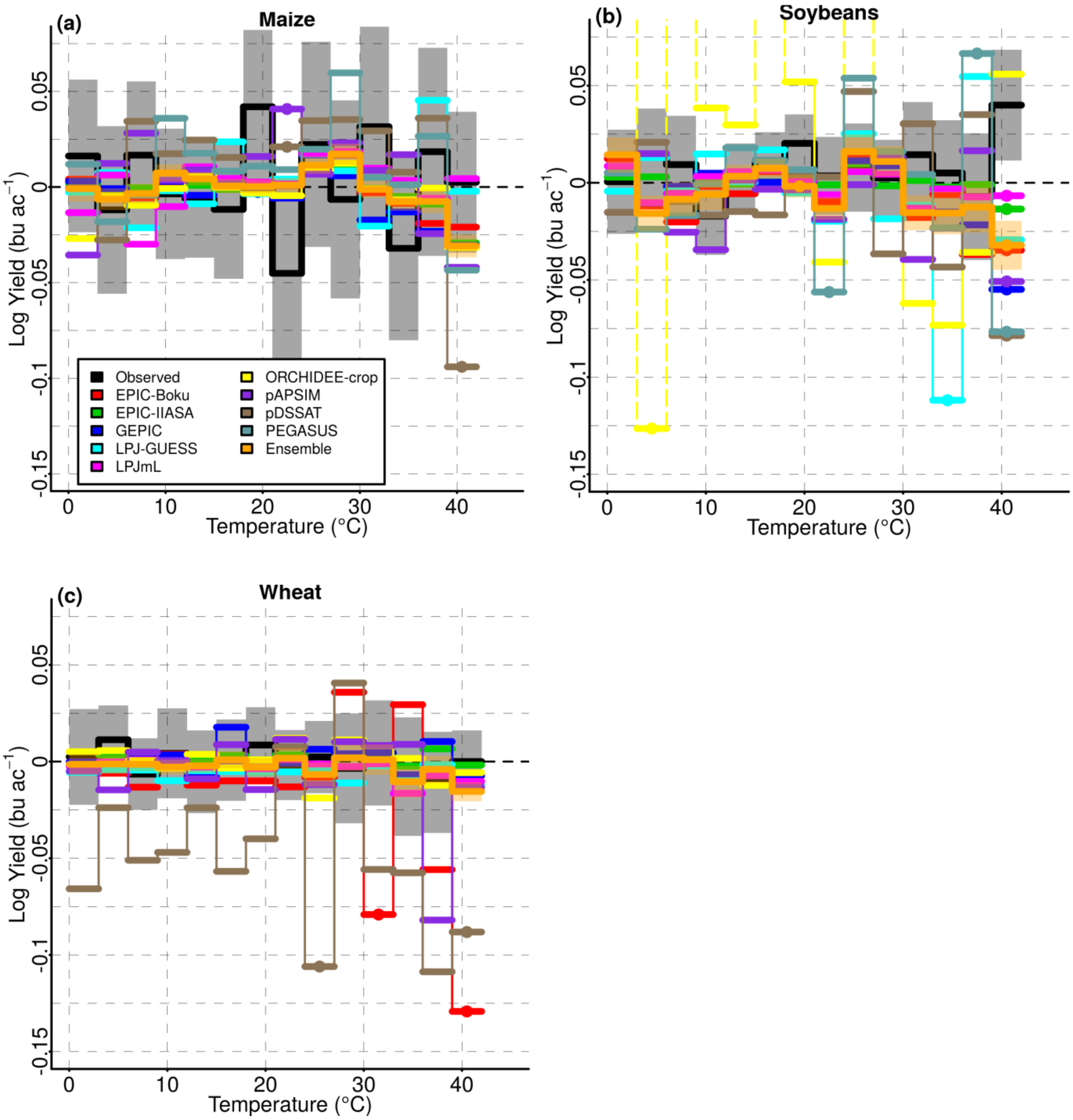

Supplementary Figure 33: Comparison of simulated to observed US yield responses to increasing temperatures for irrigated maize **(a)**, soybean **(b)** and wheat **(c)** in predominantly irrigated counties. A county is considered as predominantly irrigated if its share of irrigated agriculture exceeds 75%. Coefficients from simulated yields are marked with a dot if they significantly deviate from the observed response.

Supplementary Tables

Supplementary Table 1: Summary of basic model characteristics that could explain yield decreases under elevated temperatures. Although the models essentially consider the same effects, the mechanistic form and the parameter choices are often highly distinct between models[1, 3].

Model	Damage to enzymes/ tissues	Increasing water demand	Decreasing water supply[a]	Increasing respiration with stress	Oxidative stress (ROS)	Impaired flowering	Hastened develop-ment	Increasing root growth under water stress
EPIC-Boku	No	Yes	Yes	Yes, only T[b]	No	No	Yes	Yes
EPIC-IIASA	No	Yes	Yes	Yes, only T[b]	No	No	Yes	Yes
GEPIC	No	Yes	Yes	Yes, only T[b]	No	No	Yes	Yes
LPJ-GUESS	No	Yes	Yes	Yes, only T[b]	No	No	Limited	Yes
LPJmL	No	Yes	Yes	Yes, only T[b]	No	No	Yes	Yes
ORCHIDEE-crop	No	Yes	Yes	Yes, only T[b]	No	No	Yes	Yes
pAPSIM	No	Yes	Yes	No, but RUE* decreases	No	No	Yes	No
pDSSAT	No	Yes	Yes	Soybean: Yes, only T[b] Maize/Wheat: as pAPSIM	No	No	Yes	Yes
PEGASUS	No	Yes	Yes	No, but RUE* decreases	No	Yes[4]	Yes	Yes

[a] Decreasing water supply means the long-term effect of an increasing atmospheric demand, i.e. water that is consumed by evapotranspiration now is not available from the soil later
[b] "only T" means that respiration is only influenced by temperature, but not by water supply

Supplementary Table 2: Implementation of CO_2 effects in the nine models. The effect of these implementations has been assessed in a separate study[5].

Model	CO_2 effects*
EPIC-Boku	RUE, TE
EPIC-IIASA	RUE, TE
GEPIC	RUE, TE
LPJ-GUESS	LF, SC
LPJmL	LF, SC
ORCHIDEE-crop	LF, SC
pAPSIM	RUE, TE
pDSSAT	RUE, TE (maize, wheat), LF (soybean)
PEGASUS	RUE, TE

** LF = Leaf-level photosynthesis (via Rubisco or quantum-efficiency and leaf-photosynthesis saturation)*
RUE = Radiation use efficiency
SC = Stomatal conductance
TE = Transpiration efficiency

Supplementary Table 3: Relative differences between irrigated and rainfed AET and biomass medians for maize, soybean and wheat over the historical growing season. Differences are reported relative to the median value of the pooled samples for each crop.

Variable	Crop	Relative difference rainfed / irrigated (in %)
**AET**	Maize	14.8
	Soybean	20.0
	Wheat	21.6
**Biomass**	Maize	24.9
	Soybean	33.7
	Wheat	13.8

Supplementary Table 4: Relative differences between irrigated/rainfed and fixed present/elevated CO_2 concentrations in AET and biomass medians for maize, soybean and wheat over the future growing season. Differences are reported relative to the median value for the pairwise pooled samples. Abbreviations: rf = rainfed, ir = irrigated, CO_2- = fixed present, CO_2+ = elevated concentration.

Variable	Crop	Relative differences (in %)			
		**rf / ir with CO_2-**	**rf / ir with CO_2+**	**CO_2-/CO_2+ with ir**	**CO_2-/CO_2+ with rf**
**AET**	Maize	41.0	27.0	20.2	3.4
	Soybean	35.7	25.3	13.2	1.4
	Wheat	12.5	7.9	16.4	10.4
**Biomass**	Maize	41.0	22.8	4.6	17.1
	Soybean	41.4	16.6	35.2	43.1
	Wheat	17.8	11.2	13.5	17.2

Supplementary Table 5: Decline in length of growing season (days) for each additional degree of mean growing season temperature. Coefficients are averaged over all individual county slopes for the respective setting (*crop* x *model* x *water supply*).

Crop	Model	Rainfed	Irrigated
**Maize**	EPIC-Boku	NA[a]	NA[a]
	EPIC-IIASA	-9.1	-9.0
	GEPIC	-9.4	-9.5
	LPJ-GUESS	-9.0	-9.1
	LPJmL	-12.0	-11.4
	ORCHIDEE-crop	-3.7	-5.0
	pAPSIM	-4.6	-4.5
	pDSSAT	-7.4	-6.7
	PEGASUS	-4.0	-4.0
	Model average	***-7.4***	***-7.4***
**Soybean**	EPIC-Boku	NA[a]	NA[a]
	EPIC-IIASA	-6.3	-6.8
	GEPIC	-9.6	-9.6
	LPJ-GUESS	-5.3	-7.0
	LPJmL	-9.0	-9.4
	ORCHIDEE-crop	-3.5	-5.6
	pAPSIM	-3.5	-3.6
	pDSSAT	-2.3	-1.3
	PEGASUS	-5.6	-5.6
	Model average	***-5.6***	***-6.1***
**Wheat**	EPIC-Boku	NA[a]	NA[a]
	EPIC-IIASA	-2.6	-3.3
	GEPIC	-6.1	-4.4
	LPJ-GUESS	-1.8	-4.8
	LPJmL	3.8	-3.0
	ORCHIDEE-crop	NA	-9.0
	pAPSIM	0.5	9.5
	pDSSAT	-1.4	1.7
	Model average	***-1.3***	***-1.9***

[a] *EPIC-Boku did not provide model-specific growing seasons in the simulations used.*

Supplementary Notes

Supplementary Note 1 – Robustness of the regression approach

The regression approach does not suffer from the rather large number of explanatory variables (approx. 80 for rainfed counties). A similar response of yields to temperature can be obtained with a so-called "piecewise-linear" approach, following the ideas by Schlenker & Roberts[2], where only two temperature parameters are fitted (Supplementary Figures 1-3)). Additionally, a modified Principal-Component-Regression yields no different results than the multiple linear regression applied in the main paper (Supplementary Figure 4). This proves that multi-collinearity between the temperature exposure times is not influencing the regression results. Altogether there is ample evidence for trusting in a robust temperature response of yields in the analyzed setup, since the results do not critically depend on the regression method chosen or the number of its parameters.

The piecewise linear approach, as introduced by Schlenker & Roberts[2], performs a regression of yields against growing degree days, accumulated over the growing season. Two fixed end points at 8 and 40°C (0 and 40°C for wheat) frame the crop's response; an endogenous threshold up to which temperature affects yields positively, and above negatively, is found by looping over all possible thresholds between 15 and 35°C (maize and soybean) or 6 and 35°C (wheat) and choosing the one (threshold plus associated slopes) with the highest R^2. For more details of the method please refer to ref. [2]. This piecewise linear approach, where only two temperature-dependent slopes are estimated, exhibits the same yield response as the step-function regression applied in the main paper – which indicates that the response is stable and independent from the regression method.

A modified Principal-Component-Regression was applied to the data set to control for multicollinearity between temperature variables. We kept precipitation, county-fixed effects and state-time trends in the data matrix, but selected only those temperature bins that a principal component analysis yielded as most important (a standard deviation larger than two was used as cutoff, then representative temperature variables were selected for each component). Afterwards the standard multiple regression analysis as described in the main paper was applied to the reduced data set. For all crops the temperature coefficients are comparable to the original regression results (Supplementary Figure 4). Note that a 'classical' Principal-Component regression of *all* explanatory variables (i.e. regressing yield on transformed orthogonal components) yields similar results, but does not provide information on standard errors – this is why we resorted to the modified approach.

Supplementary Note 2 – Responses for individual models

Of the 26 crop x model cases (9 for maize, 9 for soybean, 8 for wheat) the general temperature response pattern of the rainfed observed yields is captured in 21 cases. But there are five cases where the simulated rainfed temperature response pattern strongly differs from the observed one for rainfed yields: LPJ-GUESS for maize and soybean, ORCHIDEE-crop for soybean and wheat and EPIC-Boku for wheat. The likely reason for the unexpected response is a low average yield. ORCHIDEE-crop simulates only between 34-68% of the ensemble mean yields for all three crops, LPJ-GUESS simulates 51-68% of mean yields for maize and soybean (but 117% for wheat) and EPIC-Boku simulates 67% of mean yields for wheat. The low average yields seem to reduce the signal-to-noise ratio through an increased coefficient of variation, which results in an unexpected temperature response.

Supplementary Note 3 – Coefficient correlations

To enhance visibility of coefficient differences we correlate coefficients estimated from observed and simulated yields. For each crop and irrigation setting in rainfed counties the regression coefficients γ_h from simulated yields are compared in a 1:1 plot with coefficients from observed yields. Qualitative differences between the coefficients for rainfed and irrigated yields can be seen for both maize (Supplementary Figure 13) and soybean (Supplementary Figure 15), in particular for the negative observed ones. But for wheat there is no pattern in the difference between the correlations of either rainfed or irrigated simulated yields with the observed rainfed coefficients (Supplementary Figure 14) – which confirms that there is no detectable response of historical wheat yields to high temperature. These plots are useful for telling whether there is a difference between irrigated and rainfed yield responses, for all coefficients at once rather than for single coefficients. The R^2 correlation values (in the legends) are inconclusive for the modelling capacity as there is little difference between the rainfed and the irrigated comparisons, due to the close clustering of values around 0.

Supplementary Note 4 – Model results in irrigated counties

Regression coefficients if only irrigated (fraction >75%) counties are chosen are shown in Supplementary Figure 33. There is no pattern in the response of observed yields to temperature; all coefficients (except one for maize and two for soybean) are insignificant. The yield drop at elevated temperatures above 30°C is absent in particular for maize and soybean. The positive coefficient for soybean at temperatures above 39°C may be a

regression artefact due to few days with this temperature and the insignificance of 12 of the other 13 coefficients, but does not contradict our findings. The negative responses of pDSSAT wheat (panel c, brown curve) to all except two temperature bins are insignificant (confidence intervals contain 0) and underline the independence of irrigated yields from temperature. Additionally, the sample size for irrigated wheat is small with only 10 counties in Arizona containing sufficient data. Why pDSSAT responds differently than the other models in this case has not been investigated here but would require further data on irrigated wheat.

The models generally show a slightly higher responsiveness to temperature than the observations do. This might indicate that some management decisions apart from irrigation are reflected in the observed but not in the simulated yields.

Supplementary Note 5 – Sensitivity of the regression to extreme heat

The low relative abundance of extremely high temperatures above 36°C could lead to a lower sensitivity of the statistical model to detect yield effects of these temperatures. We tested this sensitivity by artificially reducing simulated yields at each grid cell for each day above different temperature thresholds. We used 33, 36 and 39°C as thresholds, above which each day reduced crop yields by 2%. Thus, 10 days at e.g. 33°C or above reduce crop yields by a factor of $0.98^{10} = 0.817$. The reduction was additionally applied to simulated historical ensemble yields in rainfed counties. Reductions were applied to yields in grid cells and then aggregated to counties.

The statistical approach shows correct quantitative responses to artificially induced “temperature stress” by log(0.98) = -0.02 lower coefficients at and above the thresholds (Supplementary Figure 25). Thus we conclude that the regression is sensitive to extremely high temperatures, independent of their relative abundance, and that the aggregating from grid cells to counties does not conceal these events. All coefficients below the threshold temperatures are unchanged, which shows the robustness of the approach and the specificity towards temperature bins.

The distribution of exposure times differs across different parts of the historical growing season (Supplementary Figure 26). Earlier parts of the (fixed) growing season contain cooler average temperatures and less high temperature events. Most of the high (above 30°C) and extremely high (above 36°C) temperature events expectably occur in the last part of the growing season. But for maize and soybean already a substantial number of these events occur in the middle part of the growing season. For wheat high temperature events occur only in the third part. It is evident that many crops experience (extremely) high temperatures already in the middle part of the growing season. Crop anthesis dates for maize (June/July), soybean (June/July) and wheat (May) usually lie at the end of part 2 or in

part 3 of the growing season[1]. Grain filling mostly occurs in the last part, which experiences the highest temperatures. Both anthesis and grain filling are known to be very sensitive to high temperatures[6, 7, 8, 9, 10, 11, 12]. Thus, effects of extreme temperatures do not seem to be underestimated by extremely high temperatures only occurring in insensitive phases of the season. A sensitivity test towards the definition of the growing season and the timing of the exposure to high temperatures has already been performed by Schlenker & Roberts[2], resulting in qualitatively and quantitatively the same responses as for the full season.

Supplementary Note 6 – Appropriateness of the climate data

The AgMERRA[13] climate data used in this study are one order of magnitude coarser (0.5° x 0.5°) than those used by Schlenker & Roberts at a 2.5-mile resolution (about 0.04°)[2]. We decided to use the AgMERRA data instead as the GGCMs from the AgMIP ensemble were also forced by them. The temperature distribution of the fine-scale data set is slightly shifted with lower densities below about 27°C and higher densities in the temperature range from 27°C to 37°C (Supplementary Figure 29). The fine-scale climate data are constructed from monthly and daily data; this is described in the supplement of Schlenker & Roberts[2]. The comparison between the two climate data sets therefore shows differences between these, but not necessarily differences between AgMERRA and the "true" climate.

We also analyzed the spatial agreement of the two temperature distributions by comparing the numbers of days with maximum temperature above certain thresholds (30°C and 32°C) for each individual 0.5° grid cell. For each cell the days within all growing seasons (March 01 till August 31) from 1980 to 2010 above these thresholds are accumulated. Day counts for the fine-scale climate data are averaged for each 0.5° grid cell, which follows a similar consideration as in Schlenker & Roberts, but could still result in a flattening of extreme outlier values. The resulting day counts correspond closely (Supplementary Figure 30, one dot corresponds to one grid cell), with R^2 values of 94% and 91%, respectively. The AgMERRA data tend to include even more hot days than the fine-scale climate data in the very hot regions.

To test the sensitivity of the coefficients to the deviations of the temperature distributions we compare our scaling coefficients based on the AgMERRA data to the ones originally derived by Schlenker & Roberts. Both estimates for observed rainfed yields agree closely (Supplementary Figure 31), in particular also in the temperature range above 30°C. There is no hint for a significant divergence of the regression coefficients based on the higher resolution temperatures and the ones based on the AgMERRA data for both maize and

[1] http://www.usda.gov/oce/weather/pubs/Other/MWCACP/MajorWorldCropAreas.pdf ; accessed on August 23, 2016

soybean (the two crops considered by both Schlenker & Roberts and also simulated by our ensemble of GGCMs).

The rainfed yields predicted from the regression model (equation 1 in the main paper) based on the AgMERRA data agree closely with the rainfed observed yields (Supplementary Figure 32). Observed and predicted yields are plotted against mean growing season temperature for maize (panel a), soybean (panel b) and wheat (panel c). Observed yields are in red, while yields predicted by the regression model are in green.

Supplementary REFERENCES

1. Elliott J, *et al.* The Global Gridded Crop Model Intercomparison: data and modeling protocols for Phase 1 (v1.0). *Geoscientific Model Development* **8**, 261-277 (2015).

2. Schlenker W, Roberts MJ. Nonlinear temperature effects indicate severe damages to U.S. crop yields under climate change. *Proceedings of the National Academy of Sciences of the United States of America* **106**, 15594-15598 (2009).

3. Rosenzweig C, *et al.* Assessing agricultural risks of climate change in the 21st century in a global gridded crop model intercomparison. *Proceedings of the National Academy of Sciences of the United States of America* **111**, 3268-3273 (2014).

4. Deryng D, Conway D, Ramankutty N, Price J, Warren R. Global crop yield response to extreme heat stress under multiple climate change futures. *Environmental Research Letters* **9**, 034011 (2014).

5. Deryng D, *et al.* Regional disparities in the beneficial effects of rising CO2 concentrations on crop water productivity. *Nature Climate Change* **6**, 786-790 (2016).

6. Barnabas B, Jager K, Feher A. The effect of drought and heat stress on reproductive processes in cereals. *Plant, cell & environment* **31**, 11-38 (2008).

7. Farooq M, Bramley H, Palta JA, Siddique KHM. Heat Stress in Wheat during Reproductive and Grain-Filling Phases. *Critical Reviews in Plant Sciences* **30**, 491-507 (2011).

8. Grant RF, Kimball BA, Conley MM, White JW, Wall GW, Ottman MJ. Controlled Warming Effects on Wheat Growth and Yield Field Measurements and Modeling. *Agronomy Journal* **103**, 1742-1754 (2011).

9. Lobell DB, Sibley A, Ivan Ortiz-Monasterio J. Extreme heat effects on wheat senescence in India. *Nature Climate Change* **2**, 186-189 (2012).

10. Porter JR, Gawith M. Temperatures and the growth and development of wheat a review. *European Journal of Agronomy* **10**, 23-36 (1999).

11. Sanchez B, Rasmussen A, Porter JR. Temperatures and the growth and development of maize and rice: a review. *Global change biology* **20**, 408-417 (2014).

12. Wahid A, Gelani S, Ashraf M, Foolad M. Heat tolerance in plants: An overview. *Environmental and Experimental Botany* **61**, 199-223 (2007).

13. Ruane AC, Goldberg R, Chryssanthacopoulos J. Climate forcing datasets for agricultural modeling: Merged products for gap-filling and historical climate series estimation. *Agricultural and Forest Meteorology* **200**, 233-248 (2015).

14. Warszawski L, Frieler K, Huber V, Piontek F, Serdeczny O, Schewe J. The Inter-Sectoral Impact Model Intercomparison Project (ISI-MIP): project framework.

Proceedings of the National Academy of Sciences of the United States of America **111**, 3228-3232 (2014).
